# SLC25A17 inhibits autophagy to promote triple-negative breast cancer tumorigenesis by ROS-mediated JAK2/STAT3 signaling pathway

**DOI:** 10.1186/s12935-024-03270-z

**Published:** 2024-02-24

**Authors:** Haiting Zhou, Jiahao Li, Yi He, Xiaohui Xia, Junxia Liu, Huihua Xiong

**Affiliations:** 1 Department of Oncology, Tongji Hospital, Tongji Medical College, Huazhong University of Science and Technology, 1095 Jiefang Road, Wuhan, 430030 Hubei P.R. China; 2 Department of Orthopedics, Tongji Hospital, Tongji Medical College, Huazhong University of Science and Technology, Wuhan, 430030 Hubei P.R. China

**Keywords:** Breast cancer, Autophagy, Reactive oxygen species, JAK2/STAT3

## Abstract

**Background:**

SLC25A17, a peroxisomal solute carrier, has been implicated in various physiological and pathological processes. However, its precise roles and underlying mechanisms in triple-negative breast cancer (TNBC) remain incompletely understood.

**Methods:**

The expression and survival data of breast cancer were derived from TCGA and GEO databases. A variety of in vitro assays were conducted, including proliferation, apoptosis, cell cycle, migration, and invasion. Reactive oxygen species (ROS) were measured by immunofluorescence microscopy and flow cytometry. The levels of autophagy were assessed by mRFP-GFP-LC3 confocal microscopy scanning, western blotting, and electron microscopy.

**Results:**

SLC25A17 was highly expressed in breast cancer tissues, which was found to be associated with unfavorable prognosis. Functional assays demonstrated that SLC25A17 knockdown suppressed proliferation, epithelial-mesenchymal transition (EMT), migration, and invasion. Moreover, it prompted apoptosis and autophagy. On the other hand, SLC25A17 knockdown promoted autophagy through triggering ROS accumulation, which was counteracted by N-acetyl-l-cysteine (NAC). Furthermore, the pro-apoptotic effect of SLC25A17 knockdown was reversed when treated with autophagy inhibitor 3-MA in TNBC cells, suggesting that SLC25A17 knockdown-induced autophagic cell death. Mechanistically, SLC25A17 performed its function through regulation JAK2/STAT3 signaling in TNBC. In a nude mice xenograft study, SLC25A17 knockdown markedly decreased breast tumor growth and metastasis.

**Conclusion:**

SLC25A17 up-regulation may be a critical factor driving TNBC progression by modulating ROS production and autophagy. Consequently, targeting SLC25A17 could be an effective therapeutic strategy against TNBC.

**Supplementary Information:**

The online version contains supplementary material available at 10.1186/s12935-024-03270-z.

## Introduction

Breast cancer is the most commonly diagnosed malignancy in women and a prominent contributor to global cancer mortality. Triple-negative breast cancer (TNBC), accounting for approximately 15–20% of breast cancer cases, is characterized by the absence of estrogen receptor (ER), progesterone receptor (PR), and human epidermal growth factor 2 (HER2) expression [1]. TNBC demonstrates a markedly aggressive clinical progression in comparison to other subtypes of breast cancer, with an earlier age of onset, increased potential for recurrence and distant metastasis, poorer clinical outcomes, and a lack of validated targeted therapies [2]. As a result, it is absolutely crucial to identify specific molecules that play a crucial part in the development and progression of TNBC and can be targeted for potential treatments.

The mitochondrial carrier family, also known as the solute carrier family 25 (SLC25), is the largest family of solute transporters in humans, consisting of 53 members [3]. These transporters facilitate the movement of various small molecules, including organic molecules, ions, metals, and other metabolites, across biological membranes, thereby supporting fundamental cellular functions [4]. SLC25A17 has been confirmed to be located in the peroxisomal membrane. Its primary role is believed to involve the transportation of free CoA, FAD, and NAD + into peroxisomes while reciprocating with intraperoxisomally produced PAP, FMN, and AMP [5–7]. These molecules are essential for the β-oxidation of very long-chain fatty acids and the synthesis of ether phospholipids within peroxisomes, suggesting that SLC25A17 plays a critical role in peroxisome function [8, 9]. Several studies have reported the involvement of SLC25A17 in tumor progression. For instance, it has been implicated in the development of enzalutamide resistance through metabolic reprogramming and may serve as a potential therapeutic target to overcome drug resistance [10]. Genetic alterations in SLC25A17, such as copy number variation deletions, have shown a significant correlation with overall survival and relapse-free survival in neuroblastoma patients [11]. Nevertheless, the involvement of SLC25A17 in TNBC is still not fully understood. Hence, this study aims to investigate the impact of SLC25A17 on the growth of TNBC cells and shed light on the underlying molecular mechanisms involved.

In the current study, we have made an important discovery by demonstrating that SLC25A17 is upregulated in breast cancer tissues compared to adjacent tissues, which has not been reported previously. Moreover, a correlation was observed between elevated SLC25A17 expression and unfavorable prognostic outcomes. Notably, SLC25A17 knockdown hindered DNA replication, suppressed cell colony formation, and impeded motility, thereby indicating its active participation in these essential cellular processes. Furthermore, our study provided the first evidence that knockdown of SLC25A17 inhibited tumor cell growth by inducing ROS-mediated autophagy. In terms of the underlying mechanism, we observed that SLC25A17 activated the JAK2/STAT3 pathway. Collectively, these findings suggest that SLC25A17 holds significant promise as both a novel prognostic biomarker and an encouraging therapeutic target for TNBC.

## Materials and methods

### Bioinformatic analysis

The gene expression and clinical data of pan-cancers and CESC (Cervical squamous cell carcinoma) were acquired from The Cancer Genome Atlas (TCGA) (https://tcga-data.nci.nih.gov/tcga/) and Gene Expression Omnibus (GEO) (https://www.ncbi.nlm.nih.gov/geo/) databases (GSE45827, GSE29044 and GSE1456). These datasets were applied for the SLC25A17 expression and prognostic analysis. In SLC25A17 expression analysis, Willcoxon test was used to determine statistically significant differences between normal and tumor tissues. In Kaplan-Meier (K‐M) survival analysis, patients were divided into the low SLC25A17 group and high SLC25A17 group on the basis of the optimal cutoff value, which was determined using the surv_cutpoint function in the R survival package [12]. Samples in the TCGA dataset were divided into high SLC25A17 group (*n* = 835,76.4%) and low SLC25A17 group (*n* = 258, 23.6%). Samples in the GSE1456 dataset were divided into the high SLC25A17 group (*n* = 33, 20.8%) and low SLC25A17 group (*n* = 126,79.2%).

### Cell culture and reagents

The human TNBC cell lines, namely MDA-MB-231, MDA-MB-468, and BT549, along with the normal mammary epithelial cell line MCF10A, were sourced from ATCC. MDA-MB-231 and MDA-MB-468 were grown in DMEM media containing 10% FBS. BT549 was grown in a specialized medium (CM-0041, Procell, China). MCF10A was cultured in a specialized medium (CL-0525, Procell, China). All cell lines were grown at a temperature of 37 °C in a humid environment with 5% CO2.

IL-6, chloroquine (CQ), N-acetylcysteine (NAC) and 3-methyladenine (3-MA) were purchased from MCE (Shanghai, China).

### Cell transfection with lentivirus

The cells were plated in a 12-well plate with a cellular density of 1 × 10^5^ cells per well. Once the cells adhered, lentiviruses (General Biol, Anhui, China) were mixed with Polybrene (5 mg/mL, General Biol) and added to the cells. Subsequently, stably transfected cells were selected by treating them with puromycin (Biosharp Life Sciences, Hefei, China) at a concentration of 2 µg/mL for a minimum of 2 weeks.

### CCK8 and Colony forming cell assays

For CCK8 assay, 2 × 10^3^ cells were planted into 96-well plates. After the specified incubation period, CCK-8 reagent (MedChemExpress, Shanghai, China) was added for an additional two hours. For colony forming analysis, TNBC cells were incubated for two to three weeks at a density of 500 cells per well in a six-well plate, followed by fixation in 4% paraformaldehyde and staining with 0.1% crystal violet.

### EdU incorporation assay

EdU incorporation assays were carried out in accordance with the instruction of EdU Apollo®567 imaging kit (RiboBio, Guangzhou, China). 50 µM EdU solution was used to culture the cells for a period of 4 h. Subsequently, added 4% paraformaldehyde as a fixing solution and fix at room temperature for 25 min. Remove the fixative, add 0.3% Triton X-100 as a permeator, and incubate at room temperature for 10 min. Following that, the cells were incubated for 30 min using Apollo® staining solution, and the nuclei were stained using Hoechst 33342 solution.

### Apoptosis and cell cycle analysis

The Annexin V-FITC/PI apoptosis detection kit (Yeasen, Shanghai, China) was used to carry out the apoptosis assay. Following their collection, TNBC cells were stained for 15 min at 25 °C using propidium iodide (PI) and Annexin V-fluorescein isothiocyanate (FITC). To perform the cell cycle assay, TNBC cells were gathered and underwent overnight fixation in 75% ethanol at a temperature of 4 °C. Subsequently, a staining process was carried out using the PI/RNase staining buffer for a duration of 15 min. The cell cycle and apoptosis assays were conducted using CytoFlex-LX flow Cytometer (Beckman, USA).

### Wound healing and transwell assays

In wound healing examination, once the cells have reached complete fusion, the monolayer of cells is scraped using 200 µL pipette tips. The cells that had been scraped were washed with PBS. Subsequently, TNBC cells were cultured in a medium lacking of serum. At the designated time, a microscope was used to monitor the wound closure and photos were taken.

Transwell migration assays were carried out with 8.0 μm transwell chambers (Corning, NY, USA). To evaluate the cell invasion capacity, we utilized chambers filled with Matrigel (BD Science, MD, USA) in the upper chamber. The lower chamber was filled with 500 µL of culture media containing 20% FBS, while the upper chamber was filled with 100µL of cell suspension (5 × 10^4^ cells/well). Following a designated incubation period, the cells in the upper chamber were removed, and 600 µL of 4% paraformaldehyde solution was added for fixation for 25 min. The fixing solution was then discarded, and 0.1% crystal violet solution was applied for staining for 25 min. Under a microscope, pictures of the invasion and migratory cells were captured.

### Reactive oxygen species (ROS) assay

The levels of intracellular ROS were measured using Reactive Oxygen Species Assay Kit (S0033S, Beyotime, Shanghai, China). The cells were incubated with 10 mΜ DCFH-DA for 30 min at 37 °C in the absence of light, followed by three times washes. Subsequently, intracellular ROS production was measured by flow cytometry and fluorescence microscopy (Leica, MHG, Germany).

### Monodansylcadaverine (MDC) staining

The autophagosomes were detected using MDC fluorescent staining kit (C3018S, Beyotime, Shanghai, China), following the manufacturer’s instructions. Cells were cultured in 24-well plates and treated with a 1:1000 dilution of MDC for 30 min at 37 °C in the absence of light. Then, the cells underwent three consecutive washes with assay buffer. The degree of autophagy was assessed utilizing a fluorescence microscope (Leica, MHG, Germany).

### Transmission electron microscopy (TEM)

Cells were harvested and fixed using a commercially available electron microscope fixed solution (G1102, Servicebio, Wuhan, China) for 2 h at 4 °C. Subsequently, the cells were dehydrated using a series of graded ethanol concentrations (30%, 50%, 70%, 80%, 90%, 95%) and then infiltrated with propylene oxide before being embedded in an embedding medium overnight. Ultrathin sections were sliced with a Leica ultramicrotome and double-stained with uranyl acetate and lead citrate. Finally, images were acquired using a transmission electron microscope (Hitachi, Tokyo, Japan).

### Autophagic flux analysis

The cells were seeded onto confocal dishes and subsequently infected with mRFP-GFP-LC3 adenoviral particles (HanBio, Shanghai, China). After 24 h, cells were fixed in 4% paraformaldehyde for 15 min, and the nuclei were stained using a DAPI solution. Images were obtained under confocal fluorescence microscope (Eclipse Ti-E, Nikon, Japan).

### RNA extraction and real-time PCR (RT–PCR)

RNA was isolated utilizing Trizol method and subsequently reverse transcribed into complementary DNA. Subsequently, qRT-PCR analysis was carried out using the ChamQ™ SYBR® qPCR Master Mix (Vazyme, Nanjing, China) in accordance with the provided guidelines. The determination of mRNA expression was achieved using the 2^−△△Ct^ approach. The primer sequences employed in this study are presented below: *SLC25A17*: F: 5'-GGTGGTAAACACCAGACTGAATNBC-3', R: 5'-ATNBCCGAGATTCCTTCATCTNBCGA-3'; *GAPDH*: F: 5'- GTCTCCTCTGACTTCAACATNBCG-3', R: 5'- ACCACCCTGTTTNBCTGTATNBCCAA - 3'.

### Immunofluorescence staining

To perform immunofluorescence staining, the cultured cells were fixed using 4% paraformaldehyde, permeabilized with 0.5% Triton X-100, and then blocked with 3% BSA. Subsequently, the cells were incubated with antibodies against E-cadherin (1:200, 20874-1-AP, Proteintech), Vimentin (1:200, 60330-1-Ig, Proteintech), and LC3 (T55992, 1:200, Abmart) as well as the corresponding secondary antibody (Proteintech, 1:200, Wuhan, China). Afterward, the cells were stained with DAPI (Servicebio, Wuhan, China). Finally, the images were captured using a fluorescence microscope (Leica, MHG, Germany).

### Western blotting analysis

Initially, the TNBC cells were lysed and the proteins were extracted using RIPA buffer (P0013B, Beyotime). Then, the proteins were separated through 10–15% SDS-PAGE and blotted onto 0.45 μm PVDF membranes (Millipore). Subsequently, the membranes were then underwent overnight incubation at 4 °C with primary antibodies as follows: anti-β-actin antibody (1:2000, 20536-1-AP, Proteintech), anti-SLC25A17 antibody (1:1000, A14840, Abclonal), anti-STAT3 (1:1000, #9139, CST), anti- Phospho-STAT3 (1:1000, #9145, CST), anti-JAK2 (1:1000, #3230, CST), anti-Phospho-JAK2 (1:1000, #3771, CST), anti-Ecadherin (1:20000, 20874-1-AP, Proteintech), anti-Vimentin (1:20000, 60330-1-Ig, Proteintech), anti-MMP2 (1:1000, 10373-2-AP, Proteintech), anti-MMP9 (1:1000, 10375-2-AP, Proteintech), anti- Cleaved PARP (1:1000, #5625, CST), anti-Cleaved Caspase-3 (1:1000, #9664, CST), anti-LC3 (1:1000, T55992, Abmart), anti-P62 (1:5000, T55546, Abmart), anti-Beclin-1 (1:1000, T55092, Abmart), anti-Bcl-2 (1:1000, T40056, Abmart), anti-Bax (1:1000, T40051, Abmart). Following primary antibody incubation, the membranes were exposed to secondary antibodies at room temperature for 1 h. Finally, the protein bands were visualized using the West Pico Plus Chemiluminescent Substrate (Thermo Fisher Scientific).

### Immunohistochemistry (IHC)

Immunohistochemistry was conducted following the manufacturer’s instructions. In brief, slides were deparaffinized, rehydrated, subjected to staining using the primary antibodies against Ki67 (1:100, #9027, CST), Cleaved Caspase-3 (1:100, #9664, CST), Phospho-STAT3 (1:100, #9145, CST), LC3 (1:100, T55992, Abmart), SLC25A17 (1:100, A14840, Abclonal). The results were then evaluated by two professional pathologists independently.

### Animal experiments

Female BALB/c nude mice (GemPharmatech, Nanjing, China) aged six weeks were employed to establish the xenograft model under specific pathogen-free (SPF) conditions. Each mouse received a subcutaneous inoculation of 100 µL of PBS containing 5 × 10^6^ cells (MDA-MB-231 stably transfecting shNC and shSLC25A17) in the right axilla. Tumor volumes were measured every three days using digital calipers and calculated using the following formula: tumor volume (mm^3^) = 1/2 (length (mm) × (width (mm))^2^). After 24 days, the mice were sacrificed, and the tumor tissues were collected. All animal research was in compliance with the ARRIVE and was approved by the Animal Ethics Committee of Tongji Hospital, Tongji Medical College, Huazhong University of Science and Technology.

### Statistical analysis

Data from all experiments were presented as mean ± standard deviation (SD) of three or more biological replicates. Statistical analyses were performed using GraphPad Prism 8, employing Student’s *t*-test for comparing two groups and one-way ANOVA for comparing more than two groups. *P* < 0.05 was considered statistically significant.

## Results

### SLC25A17 is elevated in breast cancer tissues, and high SLC25A17 expression is associated with poor prognosis of breast cancer patients

**Fig. 1:**
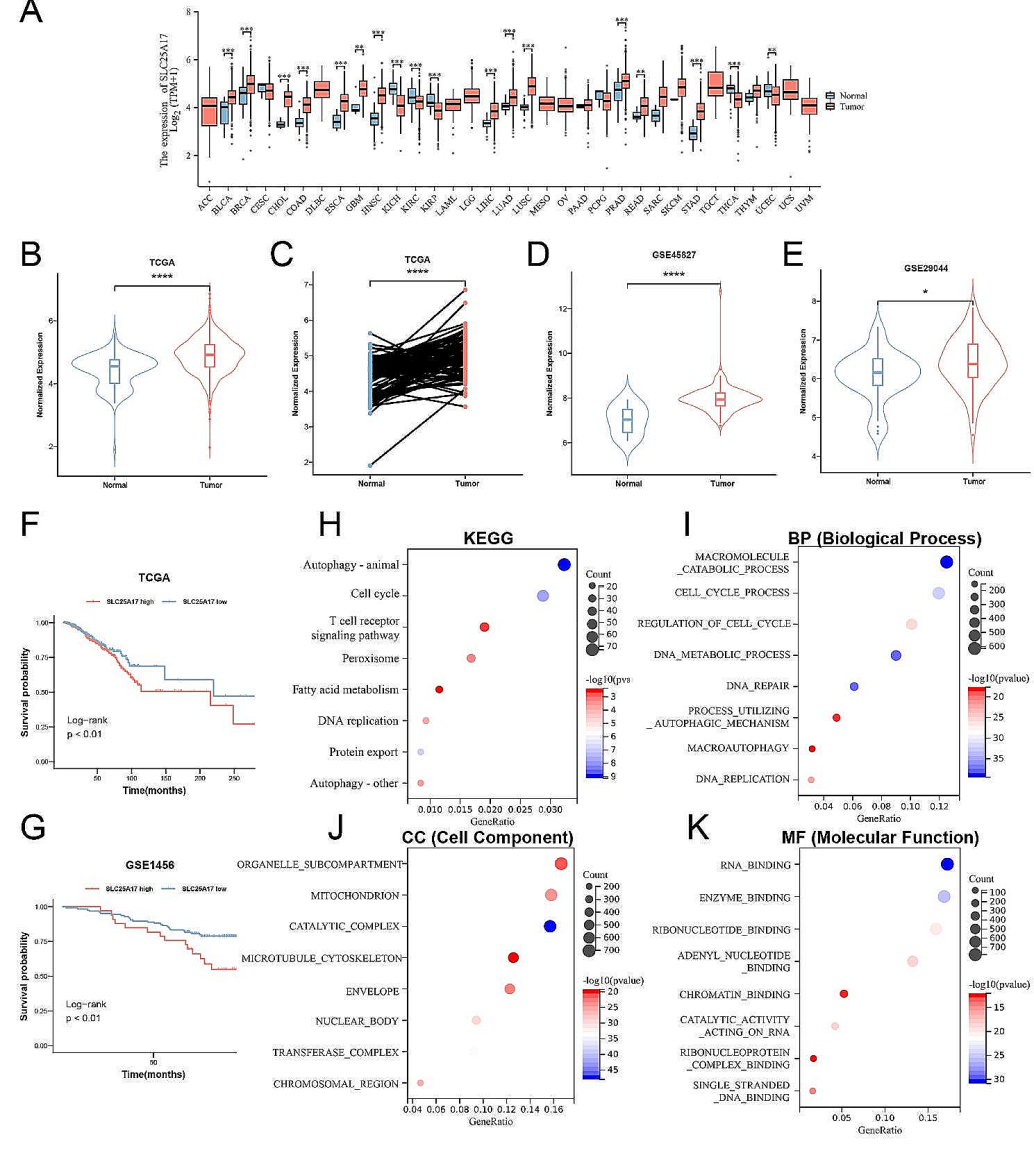
SLC25A17 was upregulated in breast cancer patients, and its high expression predicts poor prognostic. **A** SLC25A17 was aberrantly expressed among multiple cancer types. **B-E** The expression of SLC25A17 was elevated in breast cancer tissues compared to normal tissues according to TCGA, GSE45827, and GSE29044 datasets. **F, G** Kaplan–Meier analysis of overall survival probability in breast cancer patients based on TCGA and GSE1456 datasets. **H-K** Functional enrichment analysis. KEGG, biological function (BP), cell composition (CC) and molecular function (MF) were checked respectively. * *p* < 0.05, ** *p* < 0.01, *** *p* < 0.001

SLC25A17 mRNA expression levels were significantly upregulated in tumor tissues compared to normal tissues (Fig. 1A). Additionally, analysis of TCGA, GSE45827 and GSE29044 datasets revealed a significant increase in SLC25A17 expression in breast cancer tissues (Fig. 1B-E). Furthermore, higher SLC25A17 expression also correlated with a poorer overall survival (OS) in both TCGA and GSE1456 dataset (Fig. 1F, G). These findings collectively indicate upregulated SLC25A17 expression in breast cancer tissues, associated with an unfavorable prognosis.

Functional enrichment analysis demonstrated that SLC25A17-associated genes were enriched in various biological processes, including autophagy, cell cycle regulation, T cell receptor signaling pathway, peroxisome, fatty acid metabolism, DNA replication, and oxidative damage (Fig. 1H-K).

### SLC25A17 knockdown inhibits TNBC via inhibiting cell proliferation and inducing cell cycle arrest and apoptosis

**Fig. 2:**
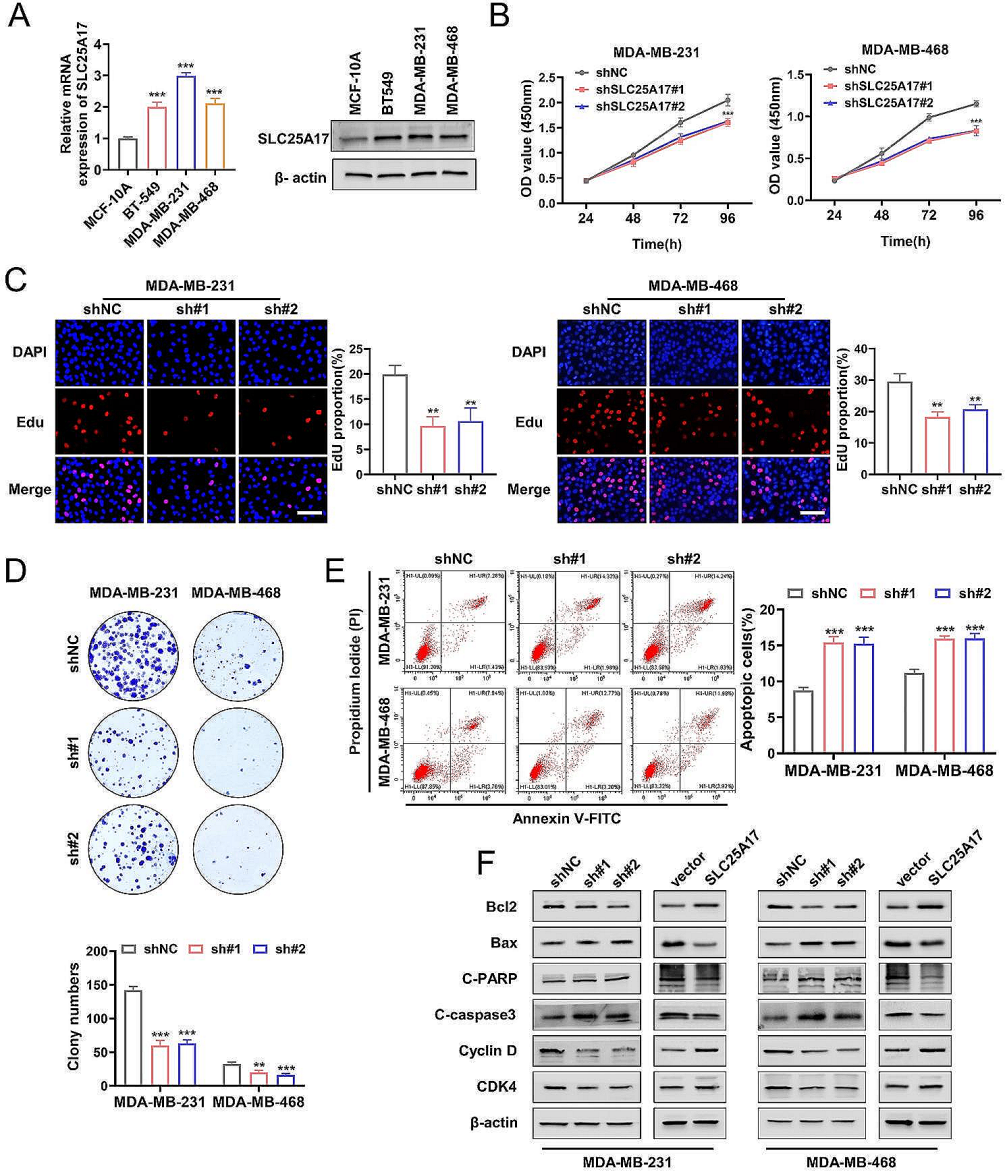
SLC25A17 knockdown inhibited TNBC cell proliferation. **A** SLC25A17 mRNA and protein expression were higher in TNBC cell lines than in breast epithelial cell. **B, C** CCK8 and EdU assays were performed to evaluate the effect of SLC25A17 knockdown on proliferative capacity in MDA-MB-231 and MDA-MB-468 cells. **D** The apoptosis level was assessed by flow cytometric analysis in MDA-MB-231 and MDA-MB-468 cells. **E** Colony formation assays were performed to evaluate the proliferative capacity in MDA-MB-231 and MDA-MB-468 cells. **F** The expression levels of apoptosis-related and cell cycle-related proteins were detected by western blot. * *p* < 0.05, ** *p* < 0.01, *** *p* < 0.001

RT-PCR and western blot analyses revealed that SLC25A17 was elevated in TNBC cell lines, when compared to the MCF10A cell lines (Fig. 2A, Additional file 1: Fig. S1A). SLC25A17 overexpression and knockdown efficacy were confirmed by RT-PCR and western blot (Additional file 1: Fig. S1B). It was observed that SLC25A17 knockdown hindered cell proliferation, whereas SLC25A17 overexpression promoted cell proliferation, as demonstrated by CCK-8 assays (Fig. 2B, Additional file 2: Fig. S2A). Additionally, EdU staining revealed a decrease in DNA replication rate in SLC25A17 knockdown cells (Fig. 2C, Additional file 2: Fig. S2B). Furthermore, colony formation assays demonstrated reduced cell colony formation following SLC25A17 knockdown (Fig. 2D, Additional file 2: Fig. S2C).

Flow cytometry revealed that SLC25A17 knockdown significantly increased apoptosis in TNBC cells, while SLC25A17 overexpression inhibited apoptosis (Fig. 2E, Additional file 2: Fig. S2D). Furthermore, cell cycle analysis revealed G1 phase arrest upon SLC25A17 knockdown (Additional file 3: Fig. S3A-B). To further confirm the effects of SLC25A17 on apoptosis and cell cycle, we analyzed markers associated with these processes. Western blot analysis corroborated the flow cytometry results, showing elevated expression of Bax, cleaved caspase-3, and cleaved PARP, along with decreased expression of Bcl-2, CDK4, and Cyclin D in SLC25A17 knockdown cells (Fig. 2F, Additional file 3: Fig. S3C). Collectively, these findings indicated that SLC25A17 knockdown led to inhibition of proliferation, promotion of G1 phase arrest and apoptosis.

### SLC25A17 knockdown suppresses cell migration, invasion and EMT in TNBC cells

**Fig. 3:**
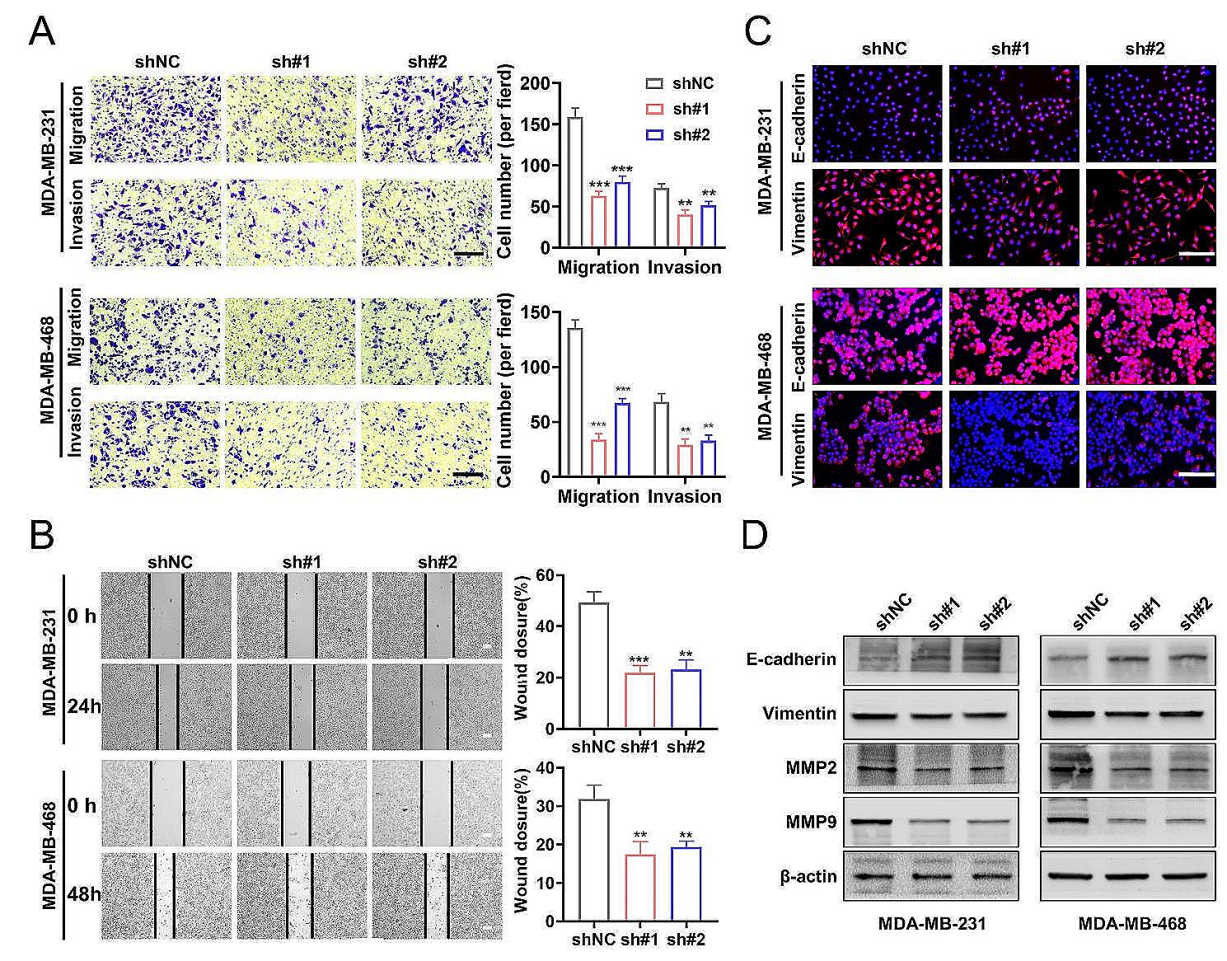
SLC25A17 knockdown inhibited migration, invasion, and EMT of TNBC cells. **A** Representative transwell migration and invasion images and statistics for the number of migrated and invaded TNBC cells with SLC25A17 knockdown. (scale bar: 100 μm). **B** Representative pictures and quantitative data of wound healing assay in MDA-MB-231 and MDA-MB-468 with SLC25A17 knockdown. **C** Immunofluorescence staining analysis of EMT markers of E-Cadherin and Vimentin (red), and the nuclei were stained with DAPI (blue) (scale bar: 100 μm). **D** Invasion-related proteins, E-Cadherin, Vimentin, MMP9 and MMP2 were detected by western blot analysis. * *p* < 0.05, ** *p* < 0.01, *** *p* < 0.001

Cell motility was assessed through transwell and wound healing assays assays. The findings indicated a decrease in migration and invasion abilities of TNBC cells following SLC25A17 knockdown, while they were enhanced after SLC25A17 overexpression (Fig. 3A-B, Additional file 4: Fig. S4A-B). Immunofluorescence assay visualized the EMT process, showing decreased Vimentin and increased E-cadherin expression in SLC25A17 knockdown MDA-MB-231 and MDA-MB-468 cells. Conversely, SLC25A17 overexpression displayed the opposite phenomenon (Fig. 3C, Additional file 4: Fig. S4C). Western blot analysis showed SLC25A17 knockdown repressed MMP2, MMP9, and vimentin, while increasing E-cadherin expression (Fig. 3D, Additional file 4: Fig. S4D-E). Collectively, these findings implied that SLC25A17 might induce EMT and promote migration and invasion in TNBC cells.

### SLC25A17 knockdown induces autophagy in TNBC cells

**Fig. 4:**
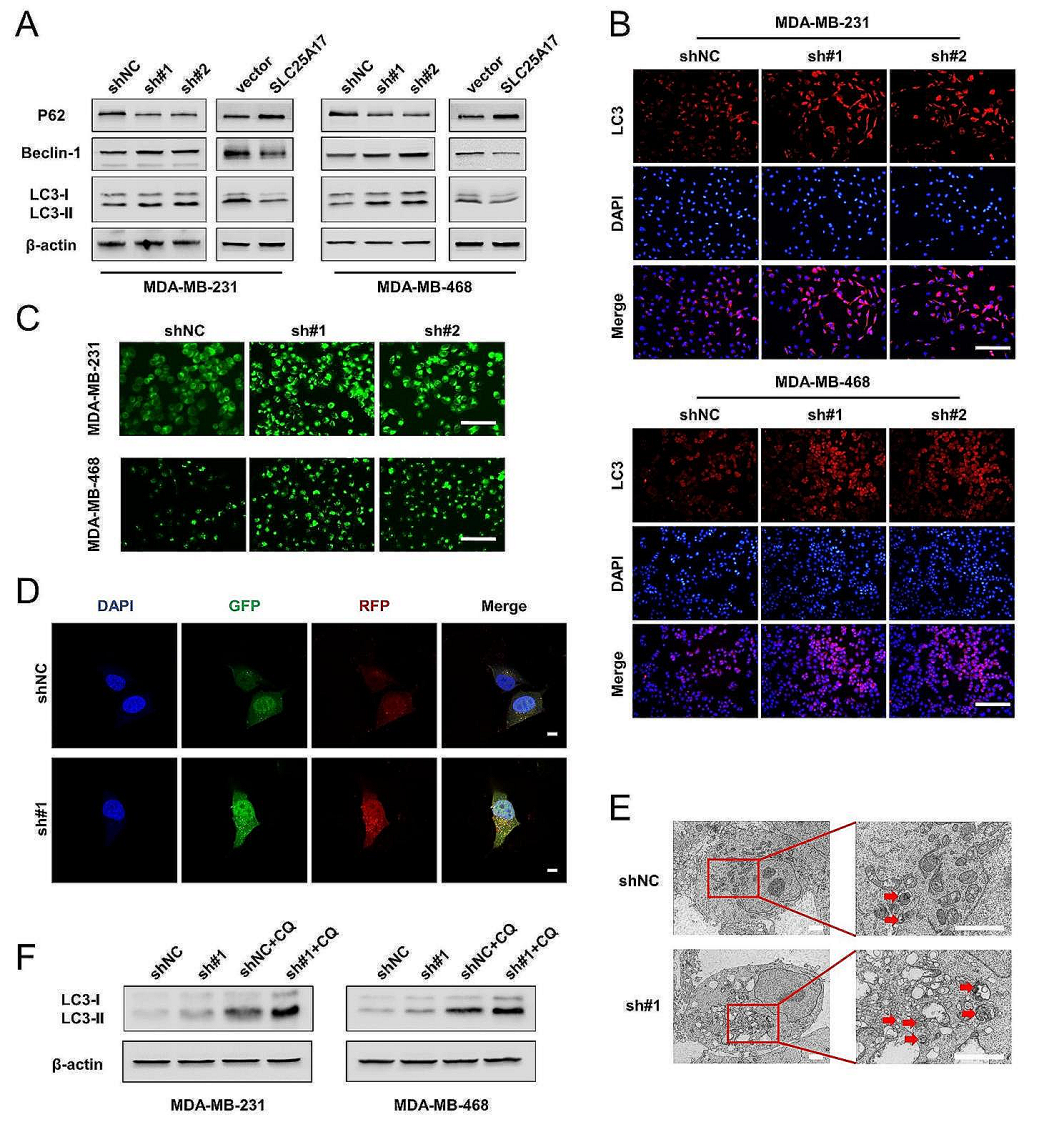
SLC25A17 knockdown induces autophagy in TNBC cells. **A** Expression levels of P62, Beclin-1 and LC3-I, LC3-II in SLC25A17 knockdown or overexpression cells were detected by western blotting. **B** Representative LC3 immunofluorescence staining using an anti-LC3 antibody in SLC25A17 knockdown cells. (scale bar: 100 μm). **C** Representative images of MDC staining for autophagosomes in SLC25A17 knockdown cells. (scale bar: 100 μm). **D** Detection of autophagic flux by the mRFP-GFP-LC3 lentivirus in SLC25A17 knockdown MDA-MB-231 cells with confocal microscopy. (scale bar: 10 μm). **E** The number of autophagosomes was detected by electron microscopy in SLC25A17 knockdown MDA-MB-231 cells, and the autophagosomes were indicated by red arrows. (scale bar: 2 μm). **F** Western blot analysis of LC3-I to LC3-II conversion in SLC25A17 knockdown cells with or without chloroquine (CQ, 20 µM)

Autophagy is a catabolic process that involves the formation of autophagosomes. It is a cellular program with dual effects, which can lead to both cell death and survival [13, 14]. LC3, P62, and Beclin-1 play crucial roles as molecular regulators and markers of autophagy [15, 16]. To investigate whether SLC25A17 knockdown induced cytotoxicity through autophagy activation, western blot analysis was performed on autophagy-related proteins. As depicted in Fig. 4A and Additional file 5: Fig. S5A, SLC25A17 knockdown significantly increased LC3-II and Beclin-1 expression, while decreasing P62 expression. Immunofluorescence analysis confirmed increased LC3 level in SLC25A17 knockdown cells (Fig. 4B, Additional file 5: Fig. S5B). Additionally, MDC staining was employed to detect autophagic vacuoles (AVs) [17]. As demonstrated in Fig. 4C and Additional file 5: Fig. S5C, SLC25A17 knockdown led to an accumulation of MDC-labeled vacuoles in the cytoplasm of TNBC cells. Then, mRFP-GFP-LC3 double-labeled adenovirus was introduced to monitor autophagy flux. It was observed that SLC25A17 knockdown increased the formation of both autophagosomes (yellow dots) and autolysosomes (remaining red dots) (Fig. 4D), indicating fusion and protein degradation within the lysosome. Furthermore, TEM results revealed that SLC25A17 knockdown markedly enhanced the abundance of autophagic double-membrane compartments with lamellar structures (Fig. 4E). Chloroquine (CQ), a well-known autophagy inhibitor, neutralizes the lysosomal pH and blocks cargo degradation in autophagosomes after fusion with lysosomes, thereby inhibiting autophagic flux [18]. As shown in Fig. 4F and Additional file 5: Fig. S5D, the combination of SLC25A17 knockdown and CQ led to increased levels of LC3-II compared to either SLC25A17 knockdown or CQ alone, suggesting that SLC25A17 knockdown induced an increase in autophagic flux. Overall, these findings indicated that SLC25A17 was involved in the autophagy process and that its knockdown enhanced autophagy levels in TNBC cells.

### SLC25A17 knockdown suppresses cell motility and promotes apoptosis by activating autophagy

**Fig. 5:**
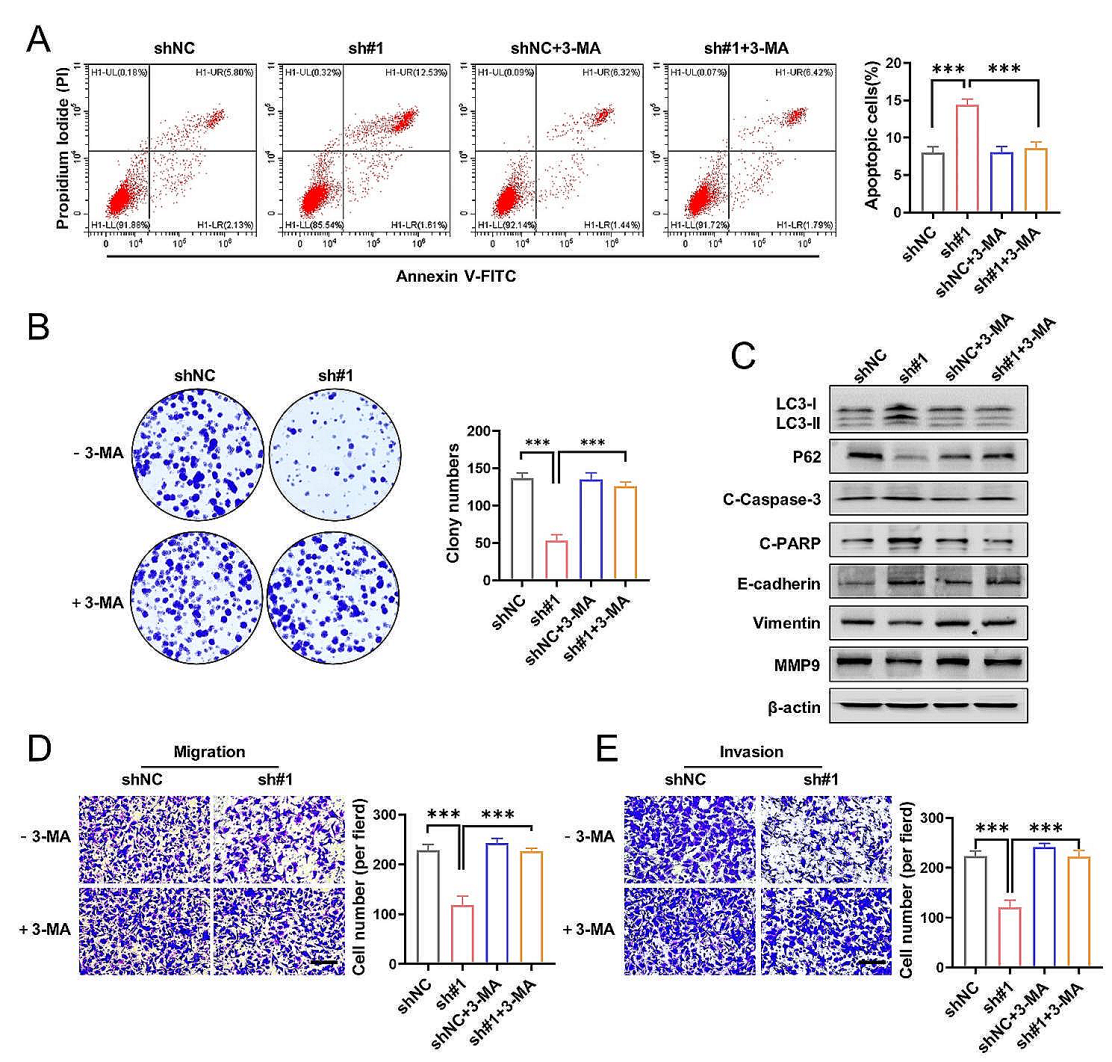
SLC25A17 knockdown inhibited motility and proliferation mediated by autophagy. **A, B** Apoptosis analysis and colony formation in SLC25A17 knockdown MDA-MB-231 cells with or without 3-MA (5 mM). 3-MA: 3-methyladenine. **C** The protein levels of LC3-I, LC3-II, P62, cleaved caspase-3, cleaved-PARP, E-cadherin, Vimentin and MMP9 were detected by western blotting in MDA-MB-231 cells. **D, E** Representative transwell migration and invasion images and statistics for the number of migrated and invaded TNBC cells in SLC25A17 knockdown MDA-MB-231 cells with or without 3-MA (5 mM). * *p* < 0.05, ** *p* < 0.01, *** *p* < 0.001

Emerging evidence has highlighted the complex interplay between autophagy and apoptosis [19, 20]. To explore the potential connection between SLC25A17 knockdown-induced apoptosis and autophagy, we pre-treated MDA-MB-231 cells with the autophagy inhibitor 3-methyladenine (3-MA) [21]. Treatment with 3-MA significantly attenuated the anti-proliferative effects of SLC25A17 knockdown, as evidenced by apoptosis analysis and colony formation assays (Fig. 5A-B). Notably, 3-MA strongly inhibited the transformation of LC3-I into LC3-II and reduced the activation of cleaved-caspase-3 and cleaved-PARP induced by SLC25A17 knockdown in MDA-MB-231 cells (Fig. 5C, Additional file 6: Fig. S6A). By migration and invasion assays, we found 3-MA blocked the anti-migratory and anti-invasive effect of SLC25A17 knockdown (Fig. 5D-E). These findings strongly suggested that enhanced apoptosis and decreased motility of TNBC cells induced by SLC25A17 knockdown is dependent on autophagy.

### SLC25A17 knockdown induces apoptosis and autophagy via ROS in TNBC cells

**Fig. 6:**
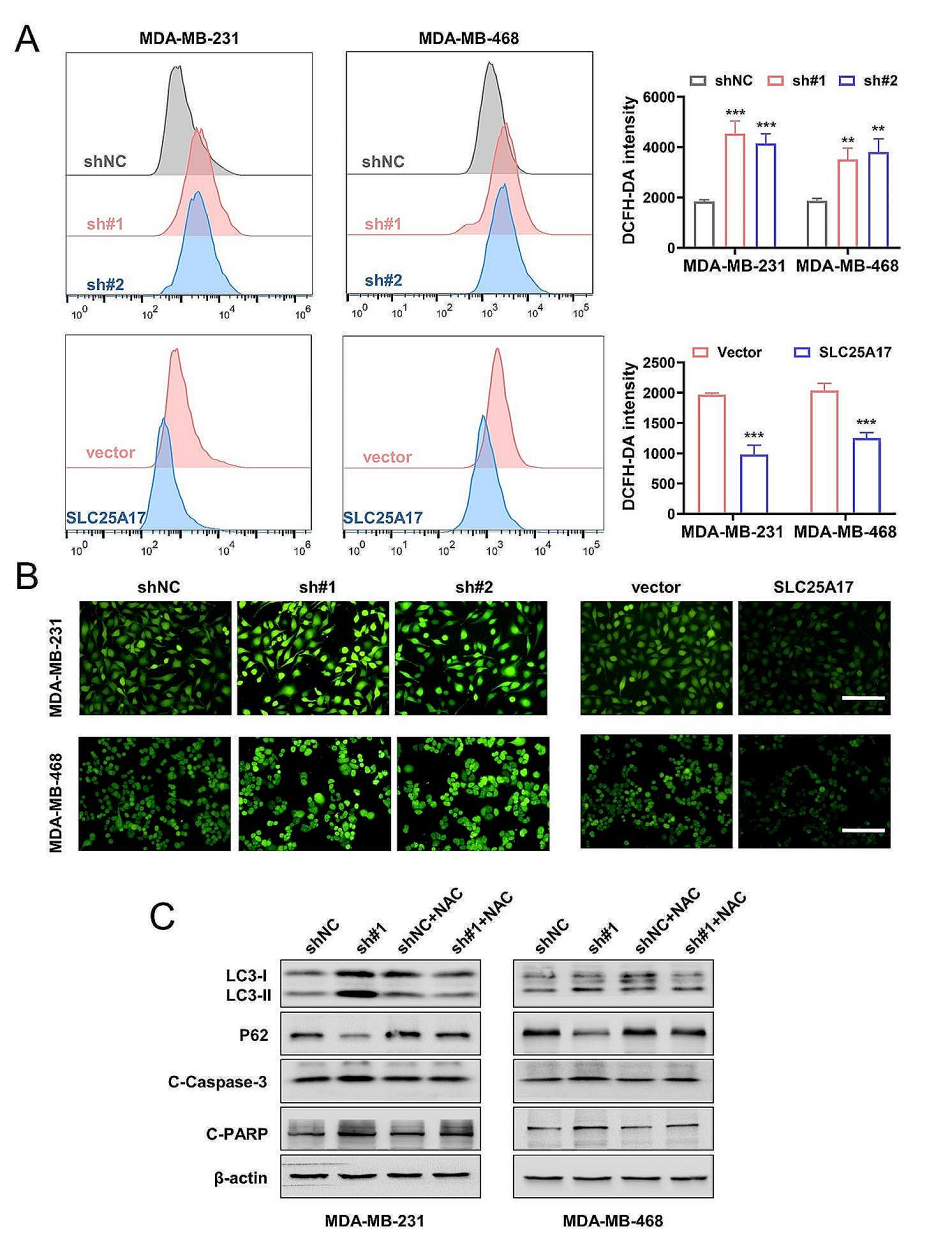
ROS induced by SLC25A17 knockdown promoted apoptosis and autophagy in TNBC cells. **A** Flow cytometric analysis and quantification of ROS production in MDA-MB-231 and MDA-MB-468 cells. **B** The level of ROS was observed by fluorescence microscopy in SLC25A17 knockdown or overexpression MDA-MB-231 and MDA-MB-468 cells. (scale bar: 100 μm). **C** The protein levels of autophagy and apoptosis-related proteins was detected by western blot in MDA-MB-231 and MDA-MB-468 cells treated with or without NAC (5 mM). * *p* < 0.05, ** *p* < 0.01, *** *p* < 0.001

Previous studies have demonstrated that the generation of ROS serves as a stimulus for inducing cell apoptosis and autophagy [22]. In order to determine whether the apoptosis and autophagy induced by SLC25A17 knockdown were a result of ROS production, DCFH-DA assay was employed to detect ROS level by fluorescent microscopy and flow cytometry in TNBC cells. The results clearly demonstrated that SLC25A17 knockdown significantly increased the fluorescent signal of DCFH-DA (Fig. 6A-B), indicating an elevation in ROS production caused by SLC25A17 knockdown in TNBC cells. Next, ROS scavengers NAC was utilized to investigate the impact of antioxidants on SLC25A17 knockdown-induced cell autophagy and apoptosis. NAC blunted the effects of SLC25A17 knockdown on autophagy and apoptosis in TNBC cells (Fig. 6C, Additional file 6: Fig. S6B). Collectively, these results indicated that SLC25A17 knockdown triggered ROS accumulation, leading to the induction of autophagy and apoptosis in TNBC cells.

### SLC25A17 knockdown induces cell apoptosis and autophagy by regulating of JAK2/STAT3 pathway in TNBC cells

**Fig. 7:**
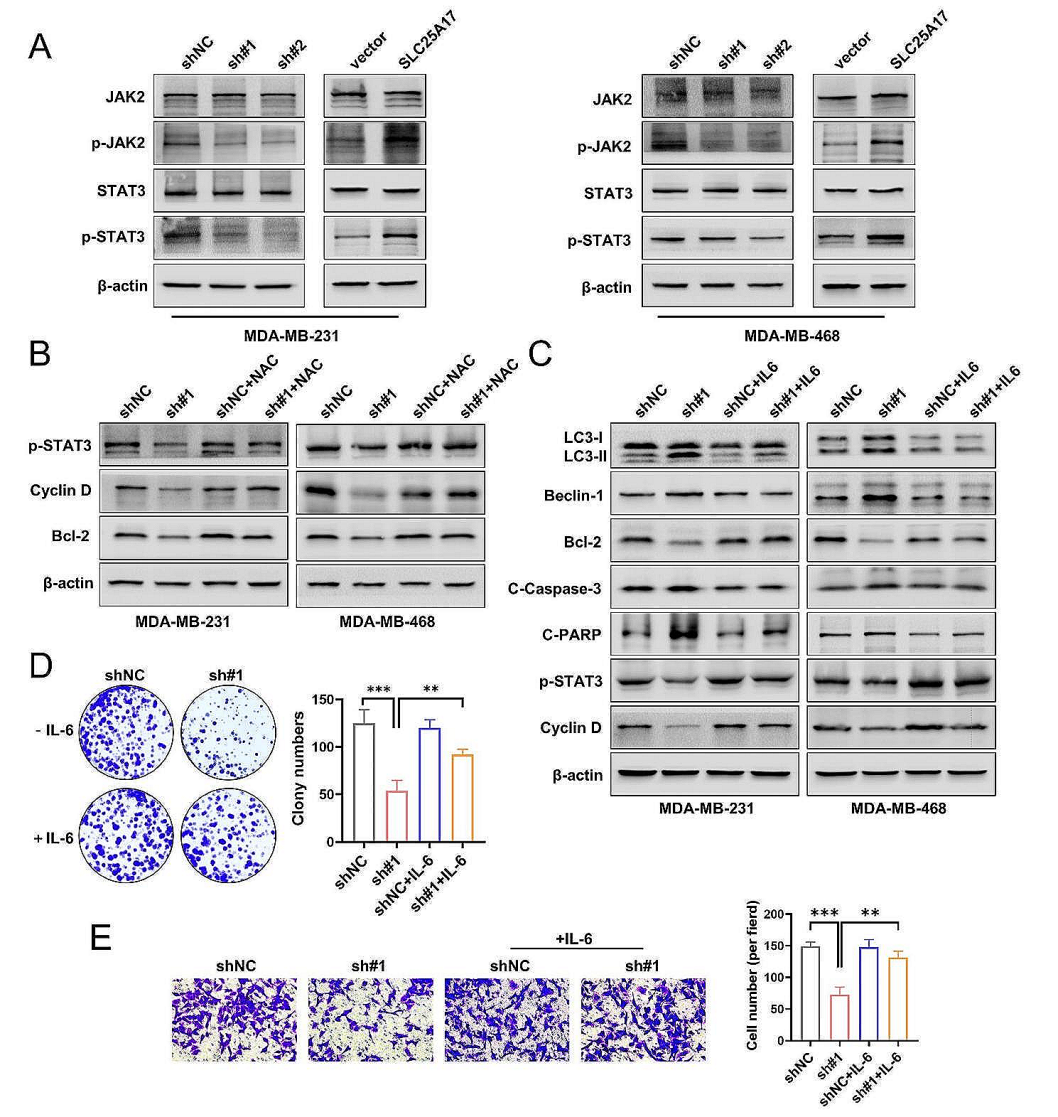
SLC25A17 knockdown inhibited JAK2/STAT3 pathway in TNBC cells. **A** The protein levels of phosphorylated STAT3 (p-STAT3) and phosphorylated JAK2 (p-JAK2) was detected by western blot in MDA-MB-231 and MDA-MB-468 cells. **B** The protein levels of p-STAT3 and its downstream protein Cyclin D and Bcl-2 were detected by western blot in MDA-MB-231 and MDA-MB-468 cells treated with or without NAC (5 mM). **C** The protein levels of autophagy and apoptosis-related proteins was detected by western blot in MDA-MB-231 and MDA-MB-468 cells treated with or without IL-6 (50 ng/mL). **D** Colony formation in SLC25A17 knockdown MDA-MB-231 cells with or without IL-6 (50 ng/mL). **E** Representative transwell invasion images and statistics for the number of invaded cells in SLC25A17 knockdown MDA-MB-231 cells with or without IL-6 (50 ng/mL). * *p* < 0.05, ** *p* < 0.01, *** *p* < 0.001

Reactive oxygen species (ROS) has been reported to inhibit the JAK2/STAT3 pathway [23]. Moreover, accumulating evidence suggests that STAT3 is a crucial regulator of autophagy [24]. Our current study demonstrated that SLC25A17 knockdown resulted in increased ROS production, followed by enhanced autophagy. Hence, our investigation focused on the potential influence of SLC25A17 on the JAK2/STAT3 pathway in TNBC cells. In our study, we observed that SLC25A17 knockdown suppressed the phosphorylation of JAK2 and STAT3, without affecting the total levels of STAT3 and JAK2 (Fig. 7A, Additional file 6: Fig. S6C). Next, we observed SLC25A17 knockdown-induced inhibition p-STAT3, and its major downstream proteins, Cyclin D and Bcl-2 were reversed by NAC, which indicated ROS are involved in the JAK2/STAT3 pathway inhibition (Fig. 7B, Additional file 6: Fig. S6D). IL-6 is a well-known cytokine that activates STAT3 signaling pathway [25]. The pro-autophagic and pro-apoptotic effects of SLC25A17 knockdown were significantly attenuated after treatment with IL-6 (Fig. 7C, Additional file 6: Fig. S6E). Furthermore, IL-6 treatment restored the impaired clone formation and invasion abilities caused by SLC25A17 knockdown in MDA-MB-231 cells (Fig. 7D-E). Collectively, the results above indicated that SLC25A17 knockdown promoted cell apoptosis and autophagy by suppressing JAK2 /STAT3 pathway, and these effects were primarily dependent on ROS production.

### SLC25A17 knockdown inhibits tumor growth in vivo

**Fig. 8:**
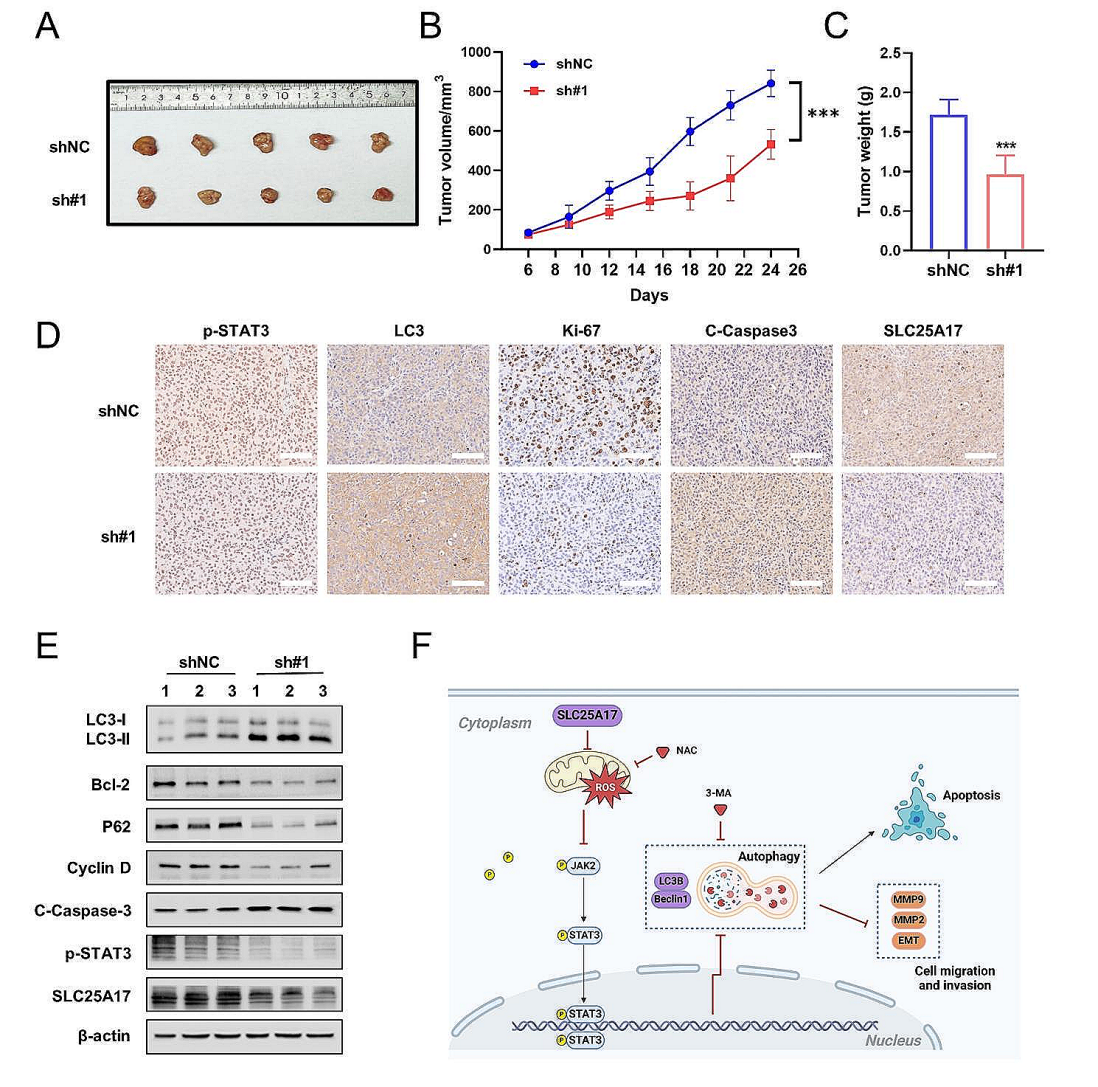
Knockdown of SLC25A17 inhibited the growth and metastasis of xenograft tumors in vivo. **A** Gross appearance of tumors from BALB/c nude mice injected with MDA-MB-231 cells with or without SLC25A17 knockdown. **B** The subcutaneous tumor volumes were tracked for 24 days. **C** Quantification of subcutaneous tumor weight of the nude mice in each group. **D** Representative IHC staining of p-STAT3, LC3, Ki-67 and cleaved-caspase3 in tumor tissues. (scale bar: 100 μm). **E** The protein level of LC3, Bcl-2, P62, Cyclin D, cleaved caspase 3 and p-STAT3 by western blot in xenograft tumors. **F** Proposed possible mechanism of SLC25A17 functions in TNBC. SLC25A17 inhibits apoptosis and autophagy by the regulation of ROS/JAK2/STAT3 signaling pathways. Image created using biorender.com. * *p* < 0.05, ** *p* < 0.01, *** *p* < 0.001

To evaluate the impact of SLC25A17 in vivo, we established a nude mouse xenograft model by subcutaneously injecting MDA-MB-231 cells transfected with sh-NC or sh-SLC25A17#1. As depicted in Fig. 8A-C, the tumors in SLC25A17 knockdown group exhibited significantly smaller volumes and lower net weights. Furthermore, immunohistochemical and western blot analyses of the xenografts demonstrated that SLC25A17 knockdown resulted in reduced expression of Ki-67 and p-STAT3, along with increased expression of cleaved caspase-3 and LC3 (Fig. 8D-E, Additional file 7: Fig. S7A). These findings provided evidence that SLC25A17 knockdown inhibited the growth of TNBC in vivo.

In conclusion, our data collectively demonstrated that SLC25A17 knockdown could induce apoptosis and autophagy through the JAK2/STAT3 pathways mediated by ROS, as illustrated in Fig. 8F.

## Discussion

The initiation and progression of TNBC are primarily driven by oncogene activation and cancer suppressor gene inactivation [26]. Therefore, it is crucial to identify potential biomarkers for early diagnosis and prognosis evaluation. In the current study, bioinformatical analysis revealed significant up-regulation of SLC25A17 in breast cancer tissues, correlating with poor prognosis. Additionally, our observations showed that silencing SLC25A17 led to suppression of cellular growth, induction of cell arrest in the G1 phase, and activation of both apoptosis and autophagy within TNBC cells. Importantly, our study uncovered the mechanisms by which SLC25A17 exerts its antitumor effects in TNBC, and demonstrated that SLC25A17 knockdown could induce apoptosis and autophagy by increasing ROS production, thereby inhibiting the activation of the JAK2/STAT3 signaling pathway.

SLC25A17 was reported to localize in the peroxisomes, and belongs to the mitochondrial solute carriers, which transports various molecules across mitochondrial membranes [27]. Several studies have reported the functions of SLC25A17 in tumors. In enzalutamide-resistant prostate cancer cells, suppression of SLC25A17 led to delayed cell cycle progression and reduced protein expression of Cyclin D1 and CDK6. Clinical analysis of prostate cancer specimens indicated patient with high SLC25A17 expression had a worse prognosis compared to those with low SLC25A17 expression [10]. Additionally, SLC25A17 exhibited elevated expression in HNSCC tissues compared to normal para-carcinoma tissues, correlating with poorer survival outcomes in patients with high SLC25A17 expression [28]. These findings were consistent with the results of our study. Here, we demonstrated that SLC25A17 was highly expressed in TNBC, and its expression was significantly correlated with the malignant biological behaviors, such as proliferation, invasion and migration. Furthermore, downregulation of SLC25A17 induced G1 phase arrest and decreased the levels of CDK4 and cyclin D1.

Autophagy is a highly conserved catabolic process in which unfolded proteins and damaged organelles are degraded through the formation of double-membrane vesicles called autophagosomes, which subsequently fuse with lysosomes for degradation [29]. Given its critical role in cancer progression, autophagy has emerged as a potential target for anticancer therapy [30]. Some studies suggested that autophagy could have a protective effect on tumor cells and promote tumor progression and drug resistance to a certain extent [31, 32]. In addition to its pro-survival effect, autophagy is also associated with a pro-death mechanism known as autophagic cell death, characterized by the sequestration of a significant portion of the cytoplasm within autophagosomes, resulting in a vacuolated appearance of the cell [33]. For example, ATG7, an essential gene for autophagy, is often down-regulated in TNBC, and its up-regulation is associated with a favorable prognosis for TNBC patients [33]. Similarly, silencing autophagic-associated protein Beclin-1 could inhibit autophagy and protect cells against death, contributing to tumor growth [34]. In our present study, we observed that downregulation of SLC25A17 promoted autophagy in TNBC cells. Treatment with the autophagy inhibitor CQ and mRFP-GFP-LC3, we observed that SLC25A17 knockdown promoted the formation of autophagosome and that the flow of autophagy was smooth. Furthermore, using the autophagy inhibitor 3-MA, we demonstrated that the pro-apoptotic effect by SLC25A17 knockdown was reversed, indicating that downregulation of SLC25A17 promoted apoptosis of TNBC cells by enhancing autophagy.

ROS plays a vital role as signaling molecules in transmitting information through various cellular signaling pathways [35, 36]. The interconnection between ROS production and autophagy regulation is well-documented in different types of cancer cells [37]. For instance, ROS-modulating natural product such as juglanin, a flavonol derived from polygonum aviculare, have been shown to induce ROS-mediated autophagy in breast cancer [38]. Overexpression of BDH2 promoted NRF2 ubiquitination and significantly induces ROS-mediated apoptosis and autophagy in gastric cancer [39]. In our study, we detected a substantial ROS production in SLC25A17 knockdown cells. However, treatment with NAC effectively rescued apoptotic cell death. Additionally, pretreatment with NAC remarkably inhibited autophagy and conversion of LC3-I to LC3-II. These findings collectively suggested that SLC25A17 knockdown induced ROS production, thereby triggering apoptosis and autophagy in TNBC cells.

Due to the complexity of the autophagy-related signaling pathway, further studies are necessary to fully understand the mechanisms underlying autophagy regulation. STAT3, a key transcription factor involved in tumorigenesis and a convergence point for multiple oncogenic pathways, plays a crucial role in tumor initiation and progression [40, 41]. Activated STAT3 has been observed in various cancers, including TNBC, where it promoted tumor cell growth, proliferation, anti-apoptosis, migration, and invasion [42]. Previous reports have showed that the JAK2/STAT3 signaling pathway could induce the expression of anti-apoptotic protein Bcl2. This disrupts the Bcl-2/Beclin-1 complex and inhibits the activation of autophagy [43, 44]. Additionally, research has shown that elevated ROS levels can inhibit tumor growth by suppressing JAK2/STAT3 signaling in different cancers [45, 46]. Thus, elevated ROS production played a crucial role in suppressing JAK2/STAT3 signaling. In our study, we observed downregulation of SLC25A17 inhibited JAK2/STAT3 pathway, and its downstream proteins Bcl-2 and Cyclin D. We then activated STAT3 using IL-6, which reversed the pro-autophagy and pro-apoptotic effects of TNBC mediated by SLC25A17 downregulation. Moreover, the inhibitory effect of SLC25A17 knockdown on JAK2/STAT3 signaling was significantly reversed by pretreatment with NAC. Collectively, our results indicated that SLC25A17 knockdown induced apoptosis and autophagy through ROS-mediated JAK2/STAT3 pathways.

## Conclusions

Our research reveals innovative perspectives on the tumorigenic role of SLC25A17 in TNBC progression, utilizing both clinical evaluation and in vivo and in vitro studies. We have demonstrated that downregulation of SLC25A17 hindered cell proliferation, prompted G1 phase arrest, and stimulated apoptosis and autophagy in TNBC cells. More importantly, we have further clarified the underlying antitumor mechanisms of SLC25A17 in TNBC and have proved that SLC25A17 knockdown could induce apoptosis and autophagy via JAK2/STAT3 pathways mediated by ROS. Altogether, these findings suggested SLC25A17 as a potentially novel therapeutic target for TNBC treatment.

### Electronic supplementary material

Below is the link to the electronic supplementary material.

Supplementary Material 1

Supplementary Material 2

Supplementary Material 3

Supplementary Material 4

Supplementary Material 5

Supplementary Material 6

Supplementary Material 7

Supplementary Material 8

## Data Availability

No datasets were generated or analysed during the current study.

## Abbreviations

TNBC: Triple-negative breast cancer
TCGA: The cancer genome atlas
ROS: Reactive oxygen species
EMT: Epithelial-mesenchymal transition
NAC: N-acetylcysteine
3-MA: 3-methyladenine
CQ: Chloroquine
MDC: Monodansylcadaverine
TEM: Transmission electron microscopy
IHC: Immunohistochemistry
OS: Overall survival
