# Supplementary figures and images for: SLC25A17 inhibits autophagy to promote triple-negative breast cancer tumorigenesis by ROS-mediated JAK2/STAT3 signaling pathway
Source: Cancer Cell Int. 2024 Feb 24;24:85. doi: 10.1186/s12935-024-03270-z (PMC10893722; doi:10.1186/s12935-024-03270-z)

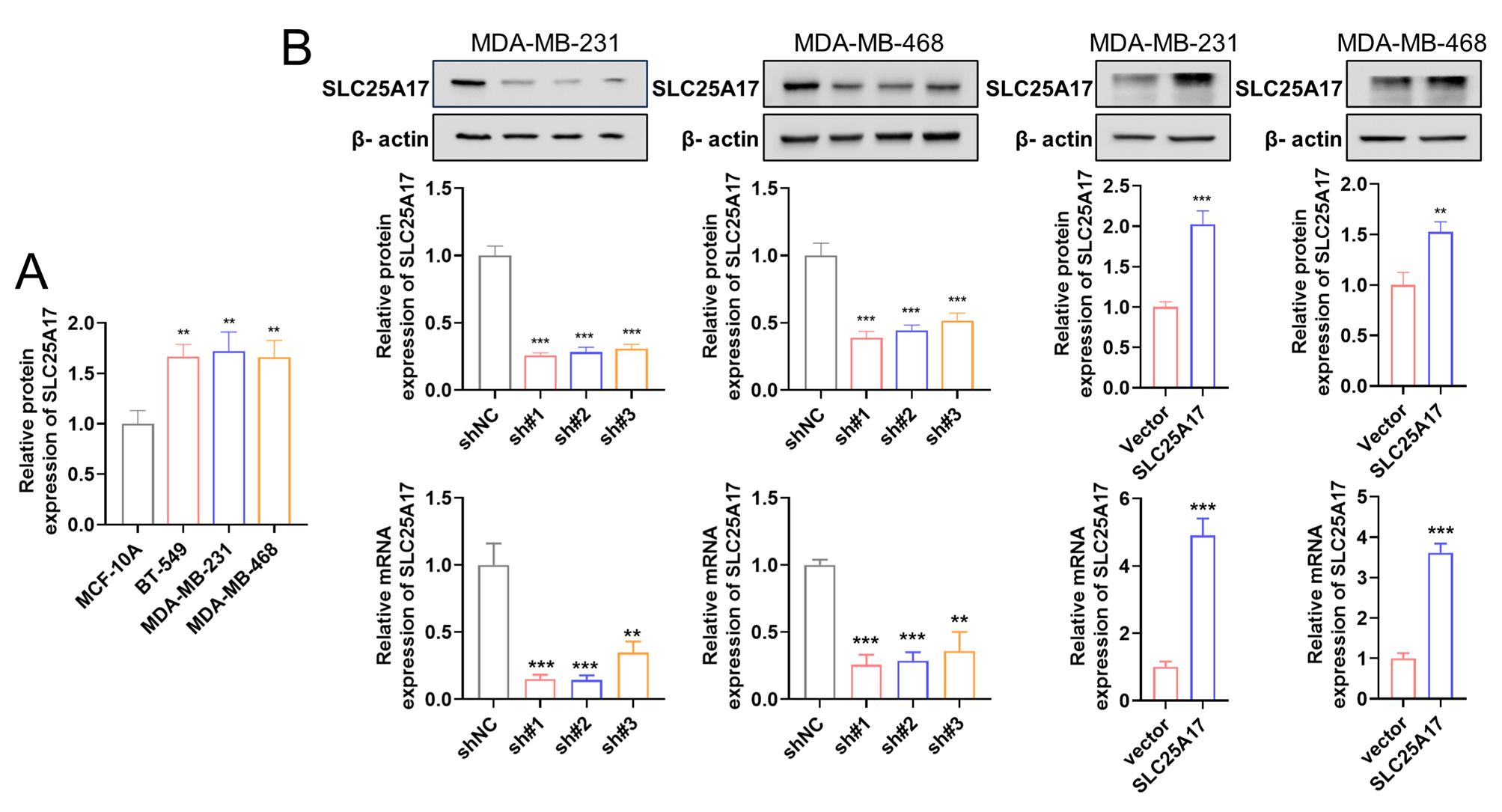

Supplement: Supplementary file 1 — Supplementary Material 1 [file 12935_2024_3270_MOESM1_ESM.tif]

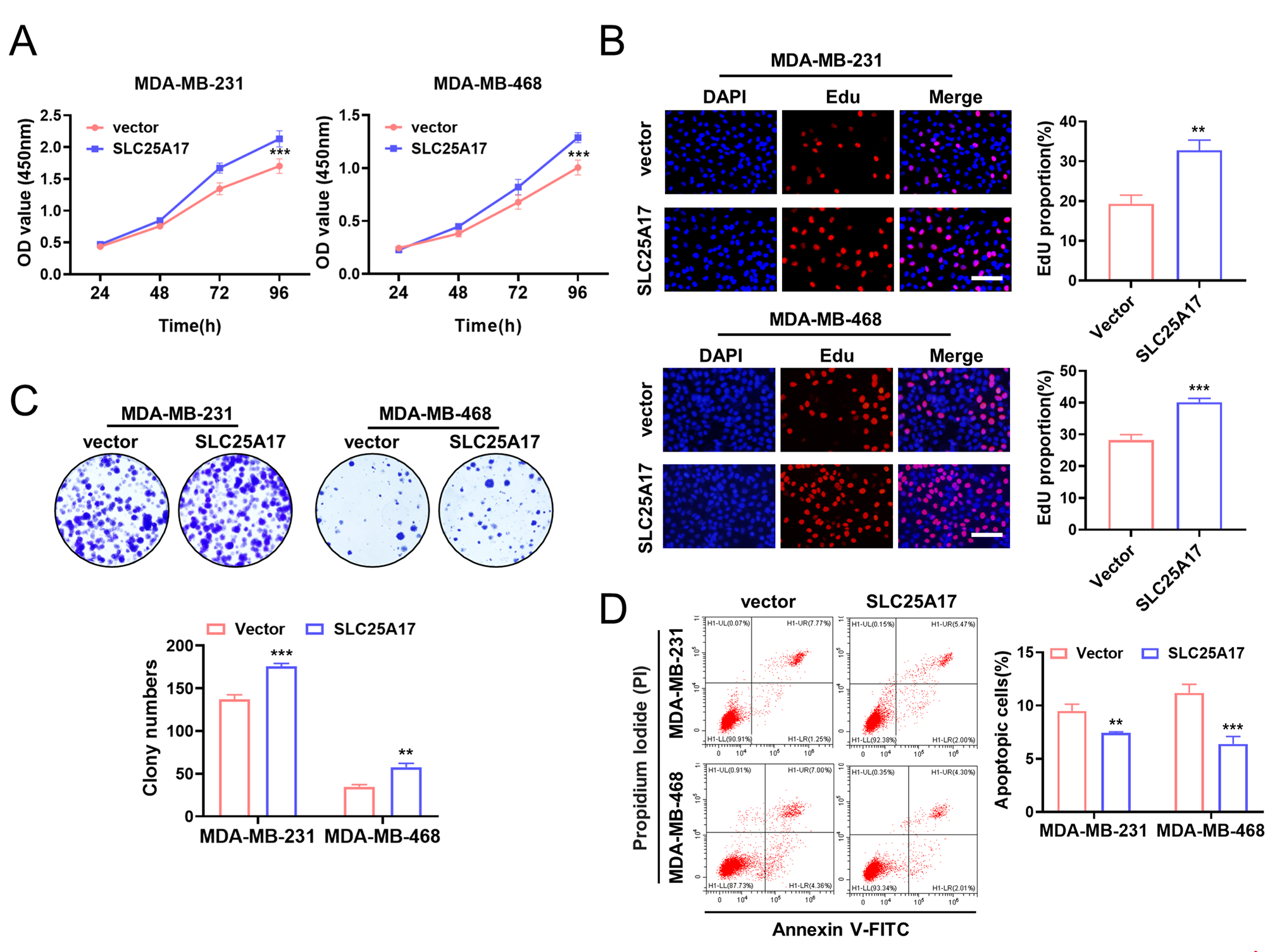

Supplement: Supplementary file 2 — Supplementary Material 2 [file 12935_2024_3270_MOESM2_ESM.tif]

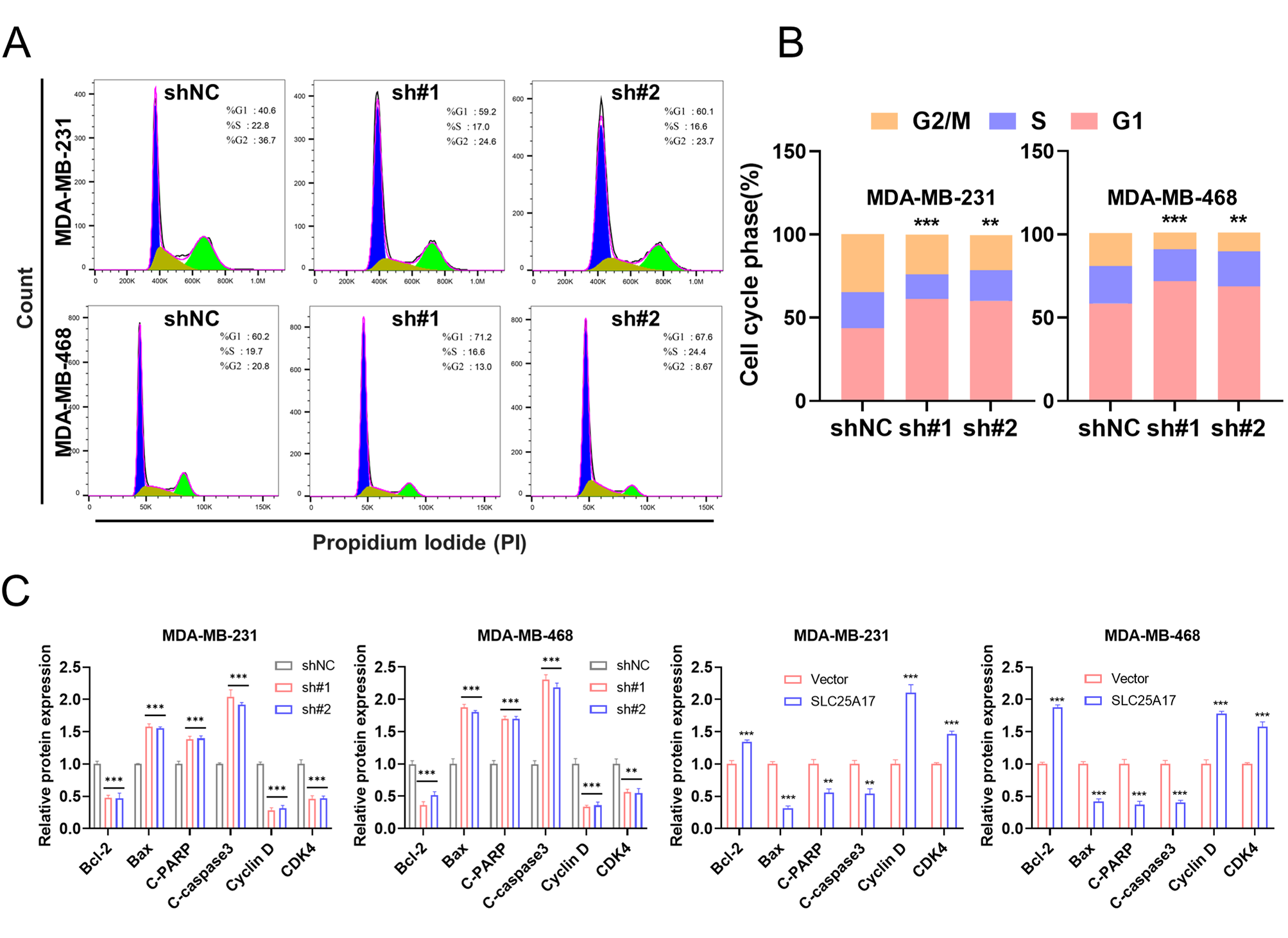

Supplement: Supplementary file 3 — Supplementary Material 3 [file 12935_2024_3270_MOESM3_ESM.tif]

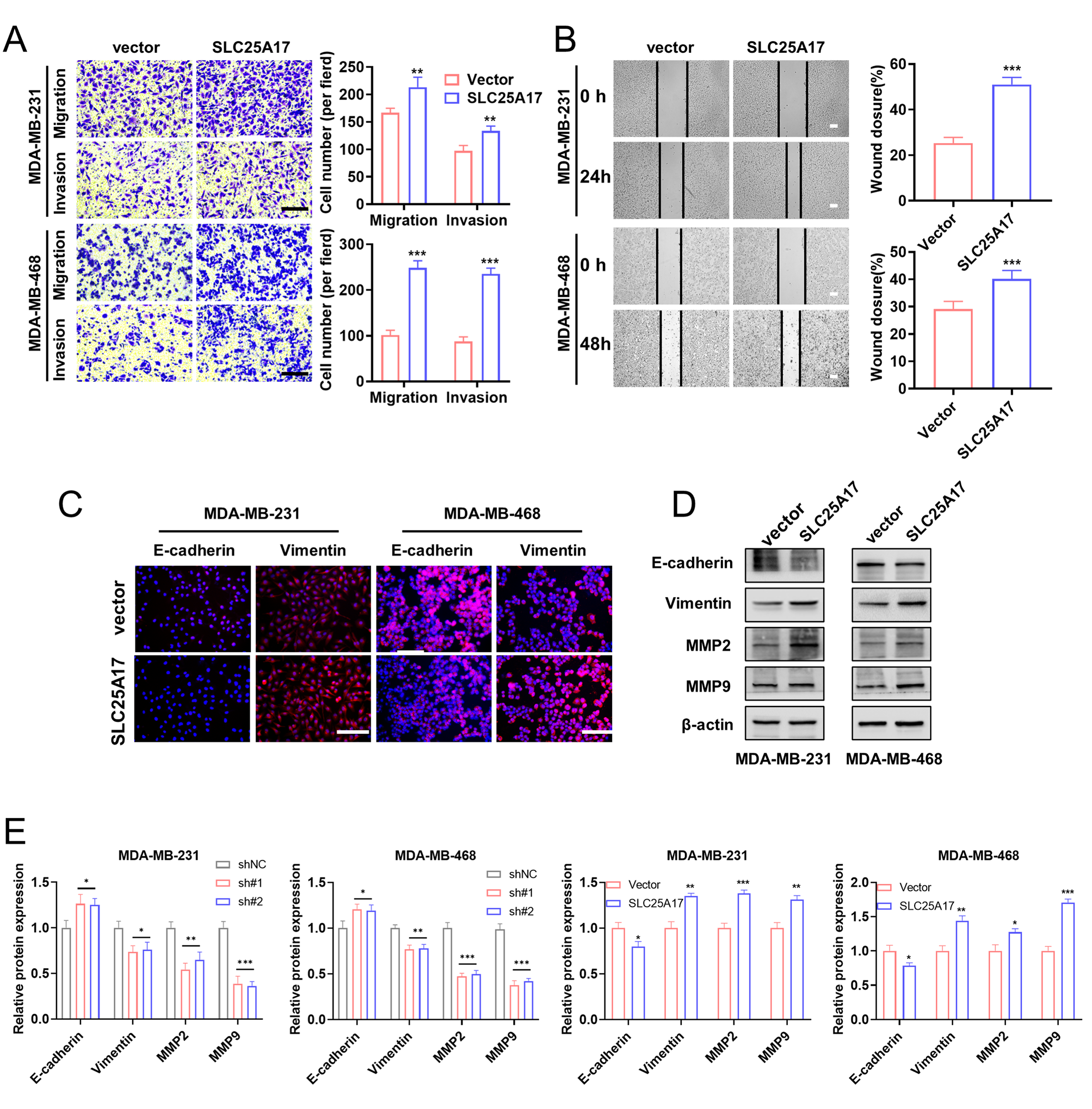

Supplement: Supplementary file 4 — Supplementary Material 4 [file 12935_2024_3270_MOESM4_ESM.tif]

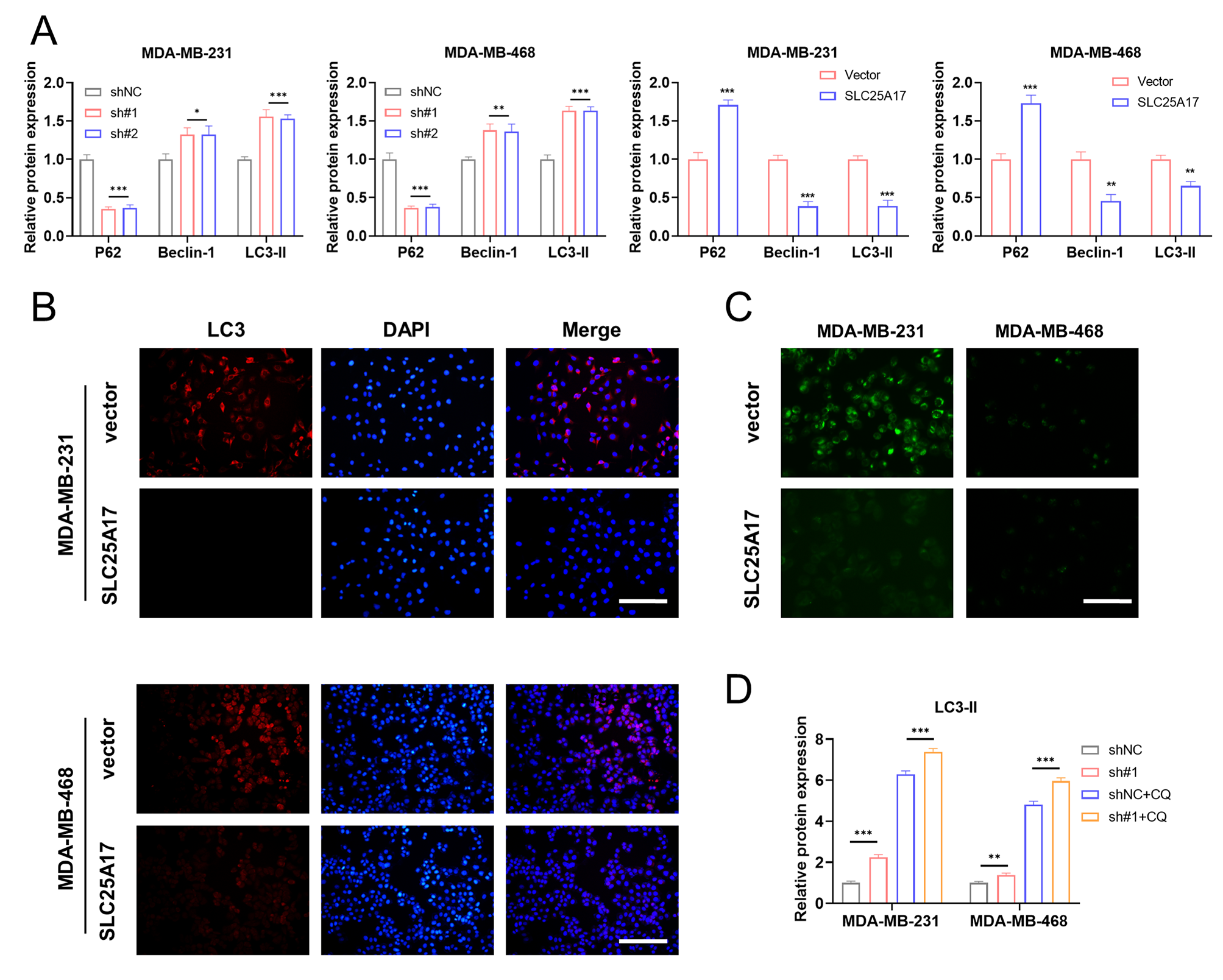

Supplement: Supplementary file 5 — Supplementary Material 5 [file 12935_2024_3270_MOESM5_ESM.tif]

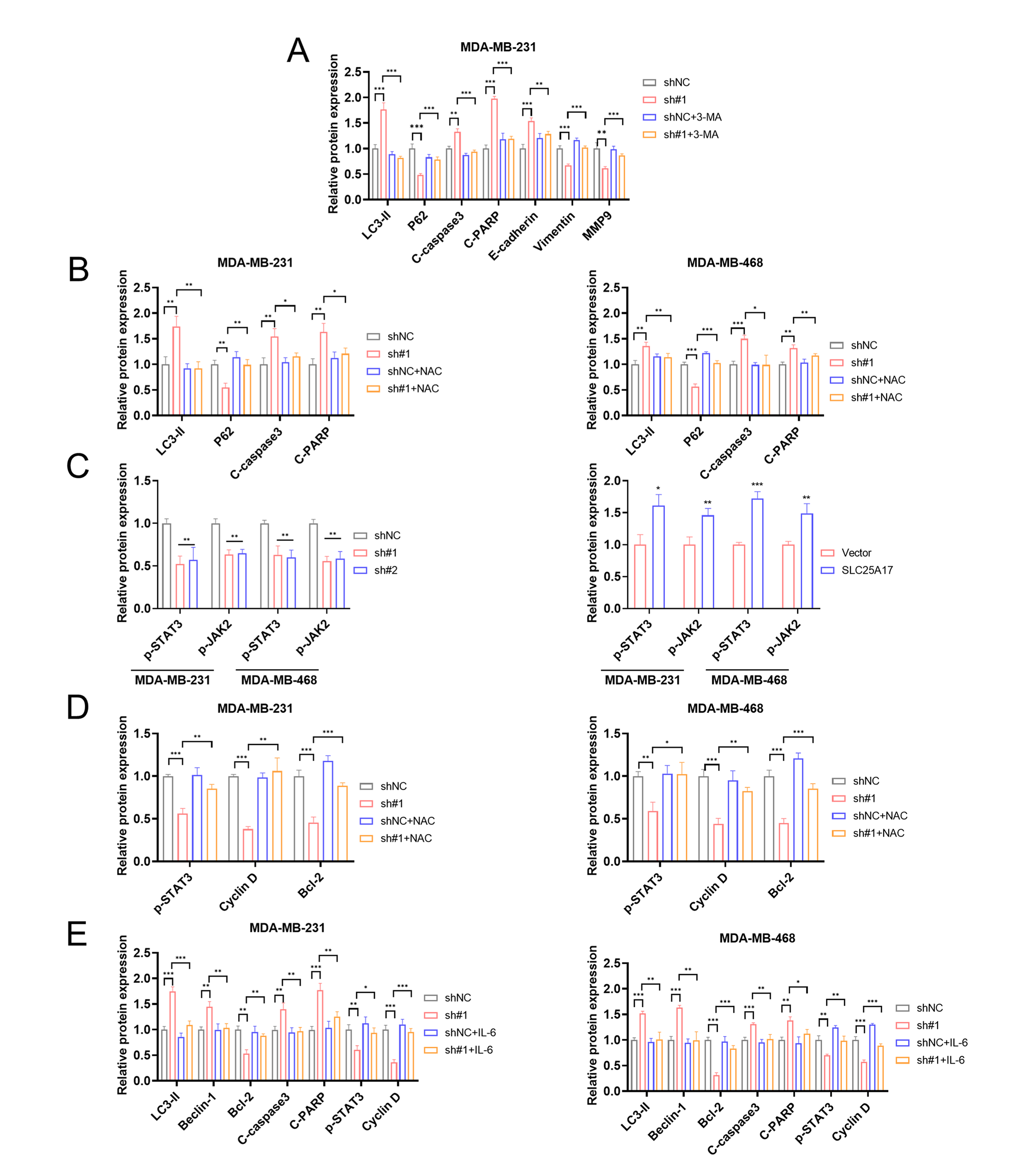

Supplement: Supplementary file 6 — Supplementary Material 6 [file 12935_2024_3270_MOESM6_ESM.tif]

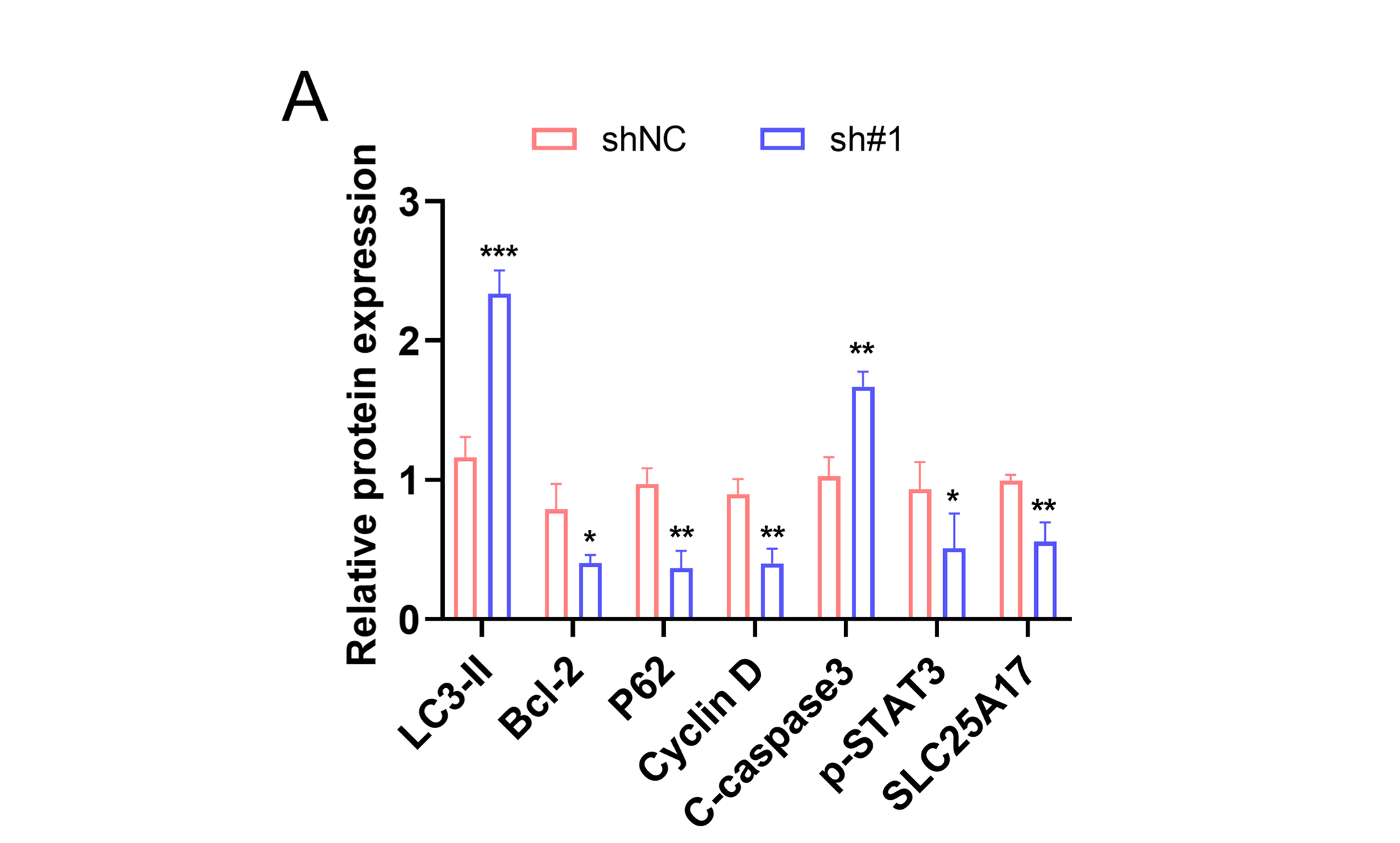

Supplement: Supplementary file 7 — Supplementary Material 7 [file 12935_2024_3270_MOESM7_ESM.tif]
