# Supplementary material for: SLC25A17 inhibits autophagy to promote triple-negative breast cancer tumorigenesis by ROS-mediated JAK2/STAT3 signaling pathway
Source: Cancer Cell Int. 2024 Feb 24;24:85. doi: 10.1186/s12935-024-03270-z (PMC10893722; doi:10.1186/s12935-024-03270-z)
Supplement: Supplementary file 8 — Supplementary Material 8 [file 12935_2024_3270_MOESM8_ESM.docx]

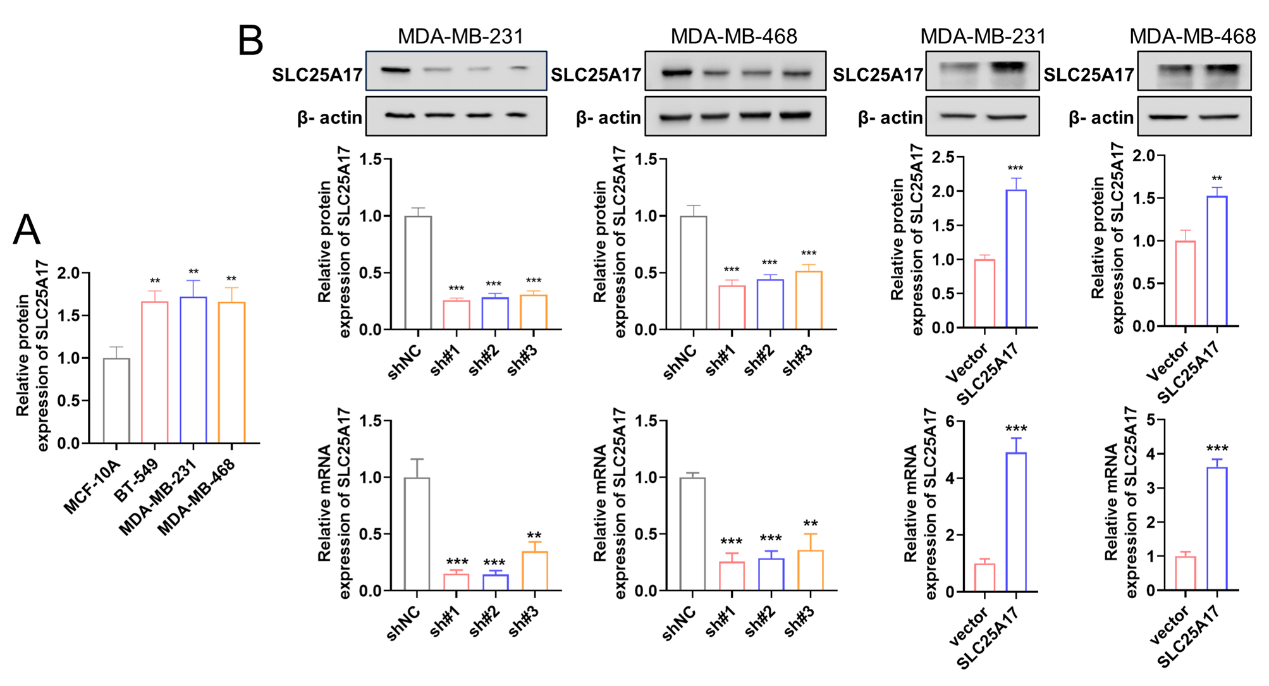


**Figure S1.** (A) A quantitative graph of SLC25A17 protein expression in TNBC cell lines and breast epithelial cell. (B) The knockdown and overexpression effect of SLC25A17 was validated by RT-PCR and western blot both in MDA-MB-231 and MDA-MB-468. * *p* < 0.05, ** *p* < 0.01, *** *p* < 0.001


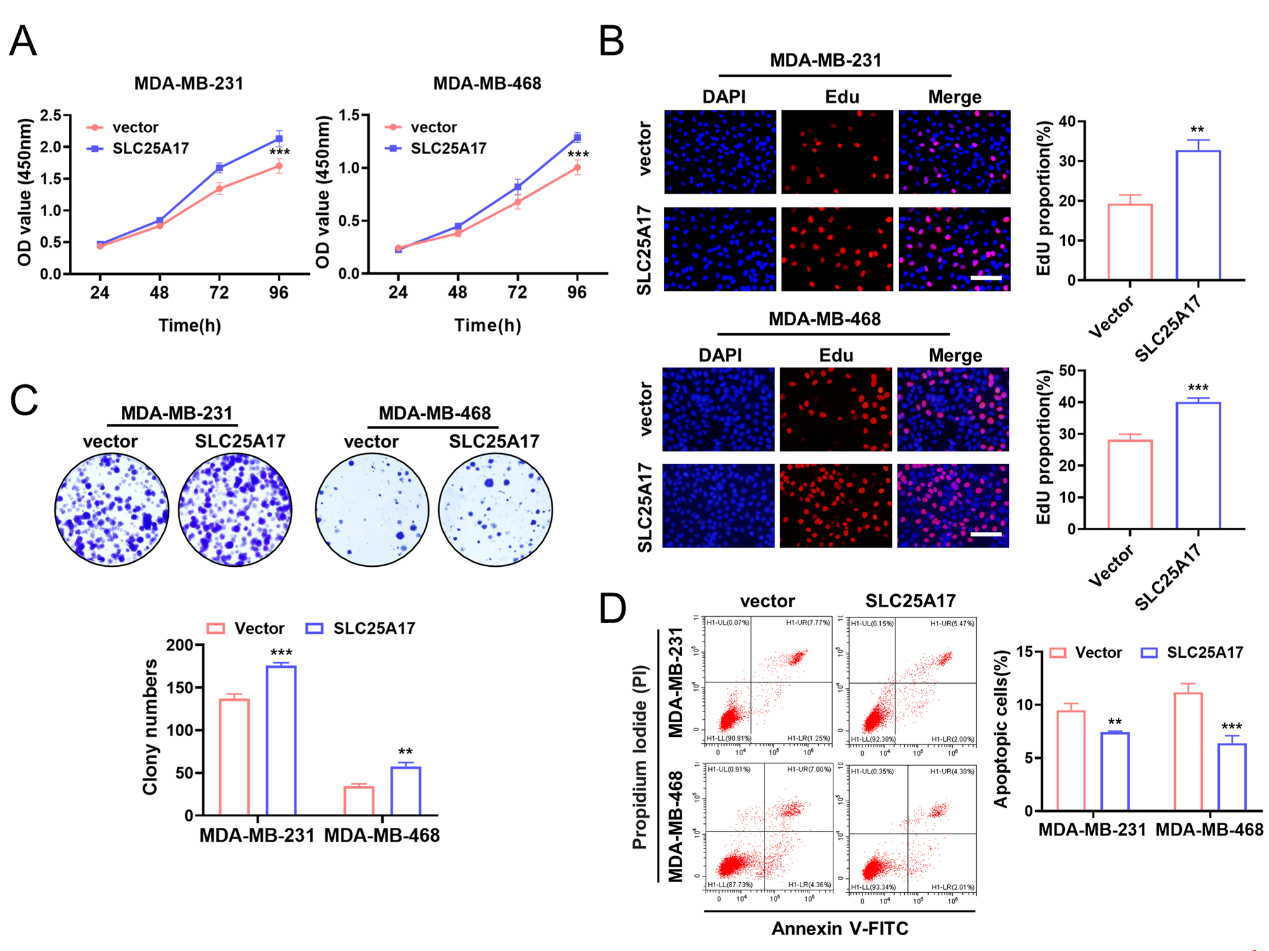


**Figure S2.** (A, B) CCK8 and EdU assays were performed to evaluate the effect of SLC25A17 overexpression on proliferative capacity in MDA-MB-231 and MDA-MB-468 cells. (C) Colony formation assays were performed to evaluate the proliferative capacity in MDA-MB-231 and MDA-MB-468 cells with SLC25A17 overexpression. (D) The apoptosis level was assessed by flow cytometric analysis in MDA-MB-231 and MDA-MB-468 cells with SLC25A17 overexpression. * *p* < 0.05, ** *p* < 0.01, *** *p* < 0.001


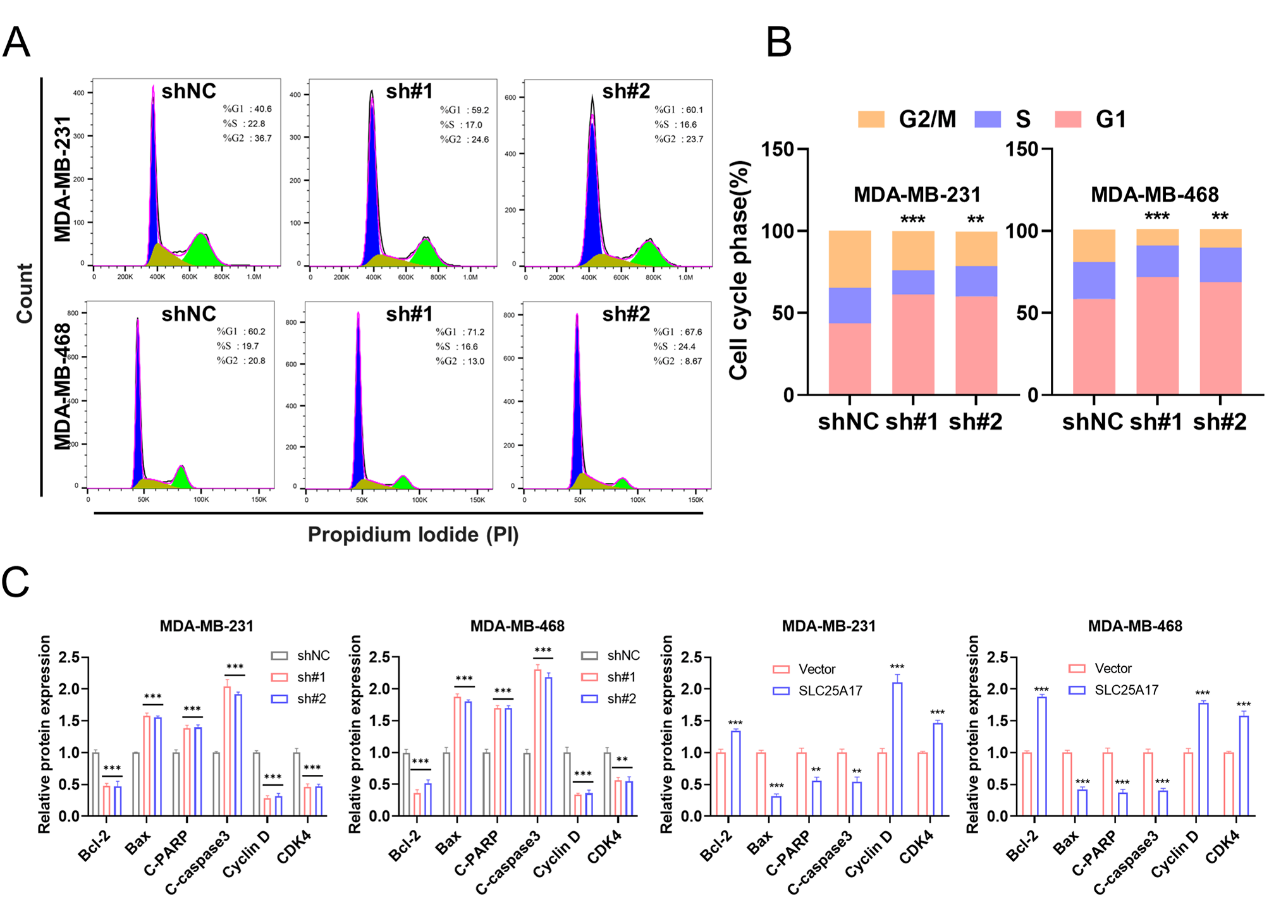


**Figure S3.** (A, B) SLC25A17 knockdown induced G1 arrest in MDA-MB-231 and MDA-MB-468 and related data statistics. (C) Quantitative data of apoptosis-related and cell cycle-related proteins after SLC25A17 knockdown and overexpression. * *p* < 0.05, ** *p* < 0.01, *** *p* < 0.001


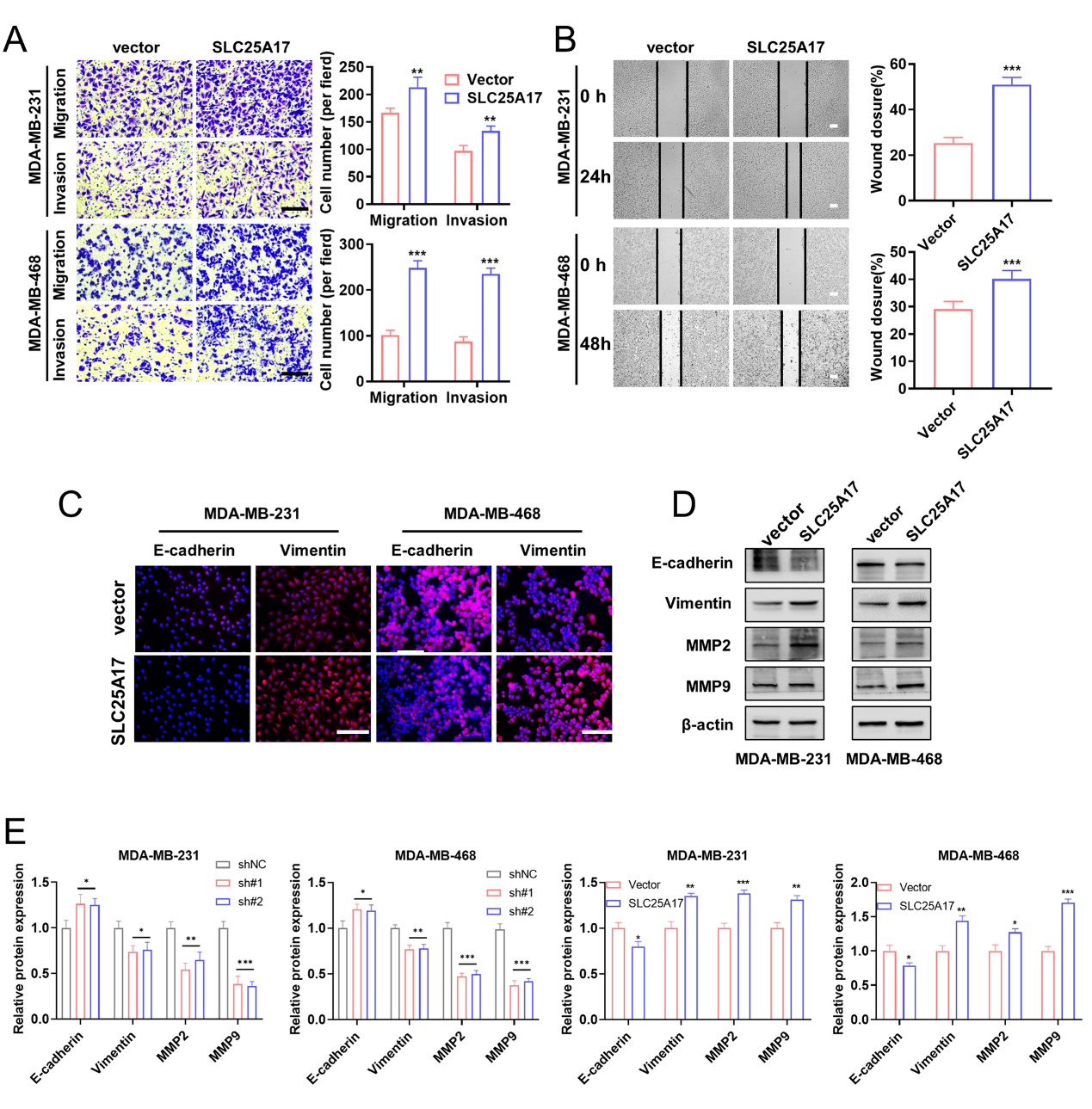


**Figure S4.** (A) Representative transwell migration and invasion images and statistics for the number of migrated and invaded TNBC cells with SLC25A17 overexpression. (scale bar: 100 μm). (B) Representative pictures and quantitative data of wound healing assay in MDA-MB-231 and MDA-MB-468 with SLC25A17 overexpression. (scale bar: 100 μm). (C) Immunofluorescence staining analysis EMT markers of E-Cadherin and Vimentin in MDA-MB-231 and MDA-MB-468 with SLC25A17 overexpression. (scale bar: 100 μm). (D) Invasion-related proteins, E-Cadherin, Vimentin, MMP9 and MMP2 were detected by western blot analysis in MDA-MB-231 and MDA-MB-468 with SLC25A17 overexpression. (E) Quantitative data of invasion-related proteins after SLC7A11 knockdown and overexpression. * *p* < 0.05, ** *p* < 0.01, *** *p* < 0.001


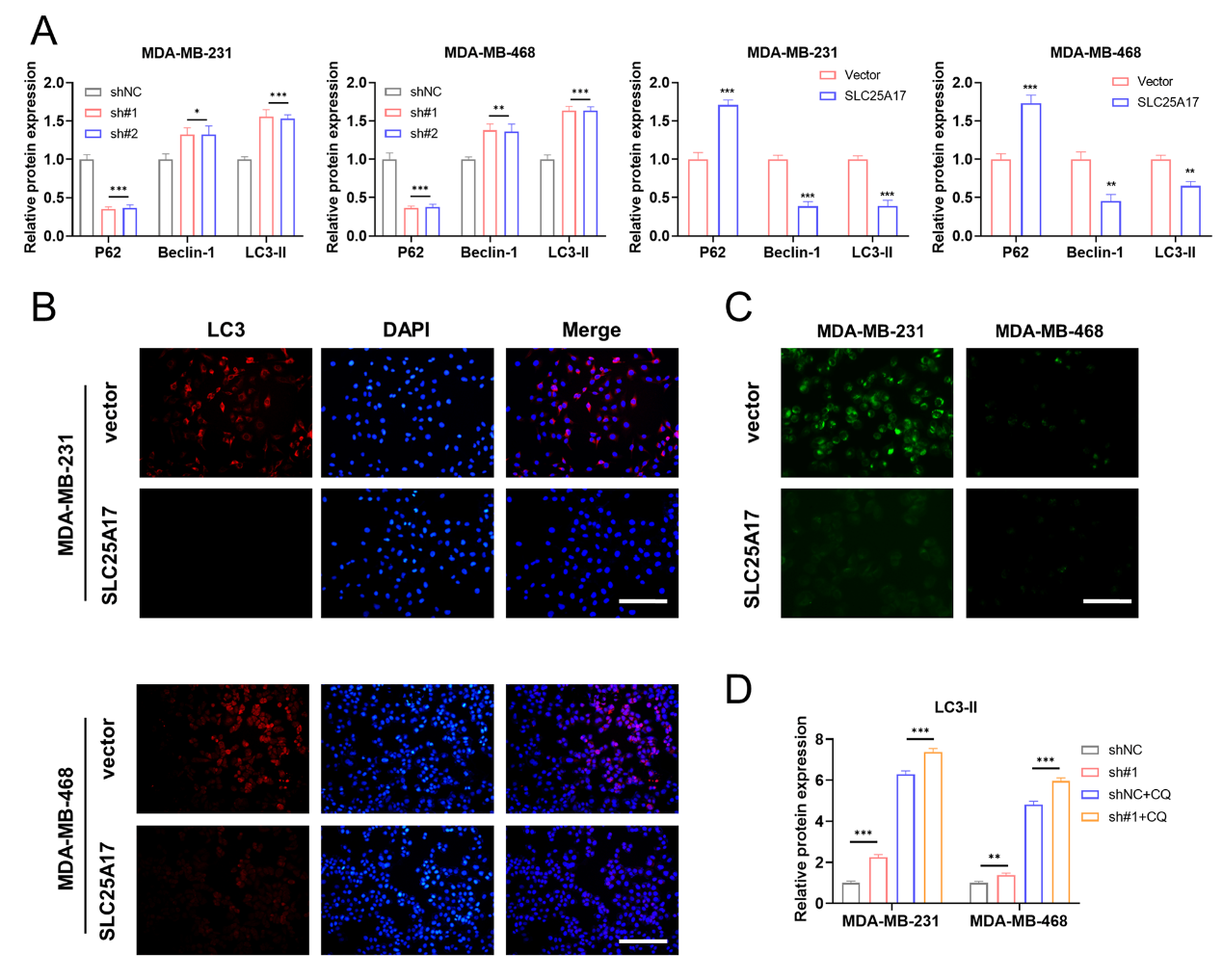


**Figure S5.** (A) Quantitative data of autophagy-related proteins after SLC7A11 knockdown and overexpression. (B) Representative LC3 immunofluorescence staining using an anti-LC3 antibody in MDA-MB-231 and MDA-MB-468 cells with SLC25A17 overexpression. (scale bar: 100 μm). (C) Representative images of MDC staining for autophagosomes in MDA-MB-231 and MDA-MB-468 cells with SLC25A17 overexpression. (scale bar: 100 μm). (D) Quantitative data of LC3-II protein in SLC25A17 knockdown cells with or without chloroquine. * *p* < 0.05, ** *p* < 0.01, *** *p* < 0.001


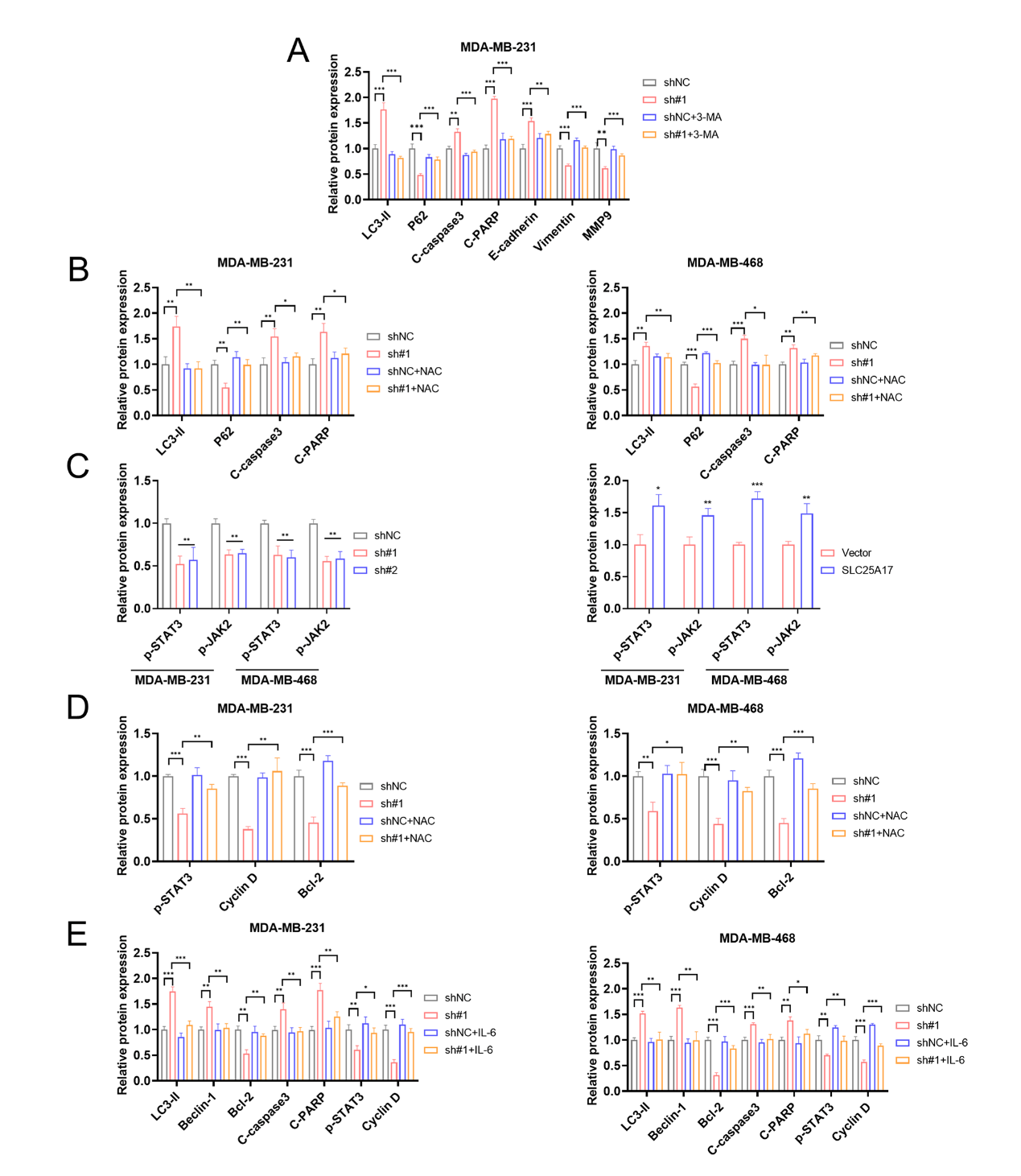


**Figure S6.** (A) Quantitative data of LC3-II, P62, cleaved caspase-3, and cleaved-PARP, E-cadherin, Vimentin and MMP9 protein level in MDA-MB-231 cells treated with or without 3-MA (5 mM). (B) Quantitative data of autophagy and apoptosis-related proteins in MDA-MB-231 and MDA-MB-468 cells treated with or without NAC (5 mM). (C) Quantitative data of phosphorylated STAT3 (p-STAT3) and phosphorylated JAK2 (p-JAK2) proteins in MDA-MB-231 and MDA-MB-468 cells. (D) Quantitative data of p-STAT3 and its downstream protein Cyclin D and Bcl-2 proteins in MDA-MB-231 and MDA-MB-468 cells treated with or without NAC (5 mM). (E) Quantitative data of autophagy and apoptosis-related proteins in MDA-MB-231 and MDA-MB-468 cells treated with or without IL-6 (50 ng/mL). * p < 0.05, ** *p* < 0.01, *** *p* < 0.001


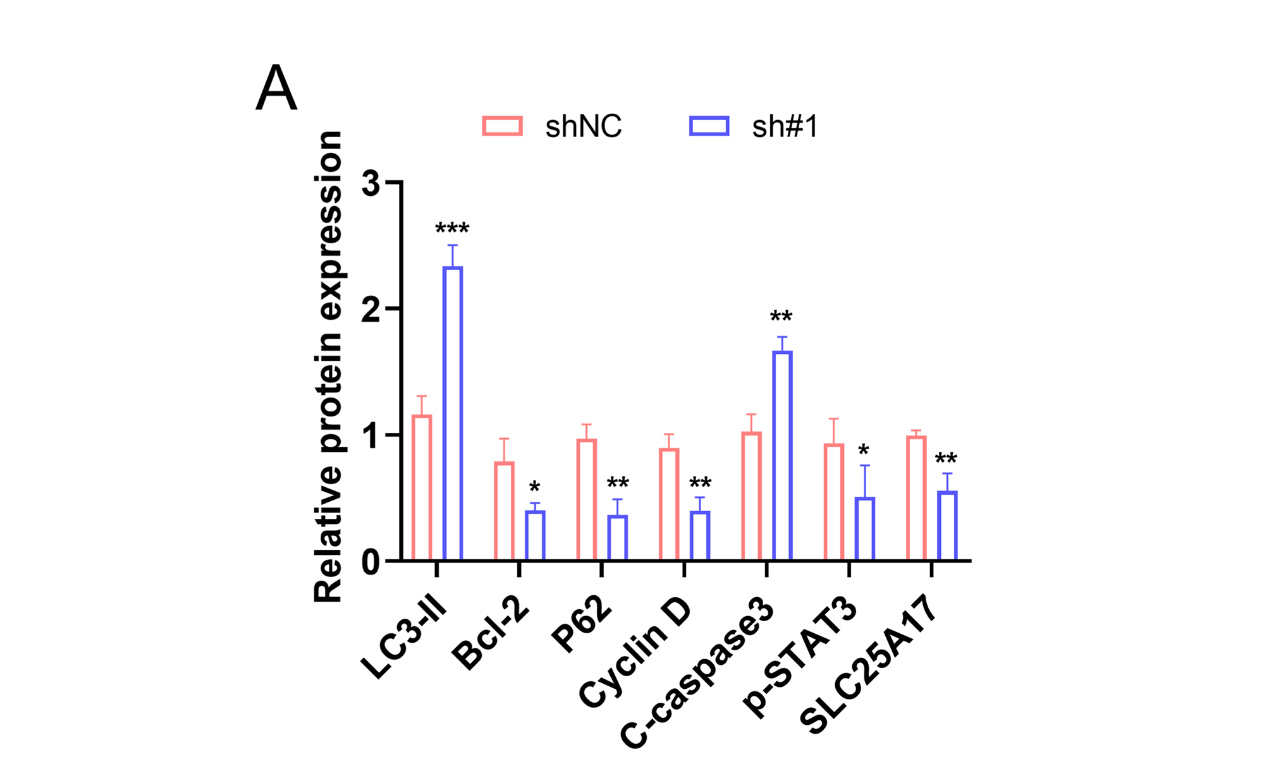


**Figure S7.** (A) Quantitative data of LC3, Bcl-2, P62, Cyclin D, cleaved caspase 3 and p-STAT3 protein level of in xenograft tumors. * *p* < 0.05, ** *p* < 0.01, *** *p* < 0.001
